# Understanding the Molecular Mechanism of miR-877-3p Could Provide Potential Biomarkers and Therapeutic Targets in Squamous Cell Carcinoma of the Cervix

**DOI:** 10.3390/cancers13071739

**Published:** 2021-04-06

**Authors:** Saioa Mendaza, Joaquín Fernández-Irigoyen, Enrique Santamaría, Imanol Arozarena, David Guerrero-Setas, Tamara Zudaire, Rosa Guarch, August Vidal, José-Santos Salas, Xavier Matias-Guiu, Karina Ausín, Carmen Gil, Rubén Hernández-Alcoceba, Esperanza Martín-Sánchez

**Affiliations:** 1 Molecular Pathology of Cancer Group, Navarrabiomed, Complejo Hospitalario de Navarra (CHN), Universidad Pública de Navarra (UPNA), Instituto de Investigación Sanitaria de Navarra (IdiSNA), Irunlarrea 3, 31008 Pamplona, Spain; smendazl@navarra.es (S.M.); dguerres@navarra.es (D.G.-S.); 2 Proteored-ISCIII, Proteomics Unit, Navarrabiomed, Complejo Hospitalario de Navarra (CHN), Universidad Pública de Navarra (UPNA), Instituto de Investigación Sanitaria de Navarra (IdiSNA), Irunlarrea 3, 31008 Pamplona, Spain; jfernani@navarra.es (J.F.-I.); esantamma@navarra.es (E.S.); kausinpe@navarra.es (K.A.); 3 Cancer Cell Signalling Group, Navarrabiomed, Complejo Hospitalario de Navarra (CHN), Universidad Pública de Navarra (UPNA), Instituto de Investigación Sanitaria de Navarra (IdiSNA), Irunlarrea 3, 31008 Pamplona, Spain; iarozarm@navarra.es; 4 Department of Pathology, Complejo Hospitalario de Navarra (CHN), Irunlarrea 3, 31008 Pamplona, Spain; tazudaire@gmail.com (T.Z.); rguarcht@navarra.es (R.G.); 5 Department of Pathology, Hospital Universitari de Bellvitge, IDIBELL, Carrer de la Feixa Llarga, 08907 L’Hospitalet de Llobregat, Spain; avidal@bellvitgehospital.cat (A.V.); fjmatiasguiu.lleida.ics@gencat.cat (X.M.-G.); 6 CIBERONC, Centro de Investigación Biomédica en Red—Cáncer, 28029 Madrid, Spain; 7 Department of Pathology, Complejo Asistencial Universitario, Altos de Nava, 24071 León, Spain; jssalas@saludcastillayleon.es; 8 Department of Pathology and Molecular Genetics, Hospital Universitari Arnau de Vilanova, University of Lleida, Alcalde Rovira Roure 80, 25198 Lleida, Spain; 9 Microbial Pathogenesis Group, Navarrabiomed, Complejo Hospitalario de Navarra (CHN), Universidad Pública de Navarra (UPNA), Instituto de Investigación Sanitaria de Navarra (IdiSNA), Irunlarrea 3, 31008 Pamplona, Spain; mariacarmen.gil@unavarra.es; 10 Gene Therapy Program, Center for Applied Medical Research (CIMA), University of Navarra, Instituto de Investigación Sanitaria de Navarra (IdiSNA), Pío XII 55, 31008 Pamplona, Spain; rubenh@unav.es

**Keywords:** miR-877-3p, ZNF177, therapeutic target, predictive biomarker, CCT3, TCP-1, cytoskeletal protein folding, squamous cell carcinoma of the cervix, high-grade squamous intraepithelial lesion, cervical cancer

## Abstract

**Simple Summary:**

Cervical cancer (CC) is managed mainly using subjective and conventional methods. Research about the molecular mechanisms of micro-RNA-877-3p (miR-877-3p) in other cancer types revealed that it interacts with events that are important for CC. Our aim was to understand the role of miR-877-3p in CC. We observed that it was overexpressed in cervical tumors compared with benign lesions, and that it promoted CC cell migration and invasion by modulating cytoskeletal protein folding, which potentiated the effects caused by paclitaxel, one of the most common therapeutic drugs used in CC. We demonstrated a functional link between miR-877-3p and one of its predicted targets, ZNF177. The expression and subcellular location of ZNF177 objectively distinguished two CC entities and predicted poor outcome in the most aggressive form. Therefore, the understanding of the molecular mechanisms driven by miR-877-3p provides useful tools for CC clinical management, currently lacking of molecular biomarkers and targeted therapies.

**Abstract:**

No therapeutic targets and molecular biomarkers are available in cervical cancer (CC) management. In other cancer types, micro-RNA-877-3p (miR-877-3p) has been associated with events relevant for CC development. Thus, we aimed to determine miR-877-3p role in CC. miR-877-3p levels were examined by quantitative-PCR in 117 cervical lesions and tumors. Effects on CC cell proliferation, migration, and invasion were evaluated upon anti-miR-877-3p transfection. miR-877-3p dependent molecular mechanism was comprehensively explored by proteomics, dual-luciferase reporter assay, western blot, and immunohistochemistry. Cervical tumors expressed higher miR-877-3p levels than benign lesions. miR-877-3p promoted CC cell migration and invasion, at least partly by modulating cytoskeletal protein folding through the chaperonin-containing T-complex protein 1 complex. Notably, miR-877-3p silencing synergized with paclitaxel. Interestingly, miR-877-3p downregulated the levels of an in silico-predicted target, ZNF177, whose expression and subcellular location significantly distinguished high-grade squamous intraepithelial lesions (HSILs) and squamous cell carcinomas of the cervix (SCCCs). Cytoplasmic ZNF177 was significantly associated with worse progression-free survival in SCCC. Our results suggest that: (i) miR-877-3p is a potential therapeutic target whose inhibition improves paclitaxel effects; (ii) the expression and location of its target ZNF177 could be diagnostic biomarkers between HSIL and SCCC; and (iii) cytoplasmic ZNF177 is a poor-prognosis biomarker in SCCC.

## 1. Introduction

According to the World Health Organization, cervical cancer (CC) is the fourth most frequent tumor type among women worldwide, and has a five-year survival of 57–67% in Europe [1,2]. This dismal outcome is partially due to it tending to be diagnosed at advanced stages, when treatments are less likely to succeed, because early phases are usually asymptomatic [2]. Additionally, at present, CC clinical management has several limitations that affect its poor prognosis: diagnosis is mainly based on the subjective interpretation of tissue morphology, there are no molecular biomarkers that can predict prognosis, and current therapies are not targeted, but based on conventional chemotherapy, radiotherapy, and surgery [2,3,4,5]. According to the European Society of Medical Oncology Clinical Practice Guidelines [2], while surgery is indicated for patients with local/locoregional disease, chemoradiotherapy involving paclitaxel and cisplatin combined with bevacizumab is considered the preferred first-line regimen in metastatic or recurrent CC, with a highest median overall survival of only 16.8 months.

These drawbacks are particularly serious in the management of two stages of CC development: high-grade squamous intraepithelial lesion (HSIL) and squamous cell carcinoma of the cervix (SCCC). A high-risk human papillomavirus (HR-HPV) infection in the cervical epithelium can lead to a benign lesion (BL). If the infection persists in dividing cells, the lesion can evolve towards an HSIL. At this stage, if some molecular alterations arise concomitantly, the tumor may invade the basement membrane of the epithelium and become an SCCC [3,6]. As tumor development is a continuous process, it is not surprising that an HSIL that has already started to invade morphologically resembles an SCCC that has just penetrated the basement membrane. HSIL patients are treated by surgery, which is successful in more than 85% of cases [7], while SCCC patients receive a more aggressive treatment that, unfortunately, is ineffective in around 50% of patients, who eventually die from the disease [2]. Since diagnosis of these entities mainly relies on the subjective evaluation of tissue morphology, some reports have evidenced the need of objectively measurable strategies to distinguish HSIL and SCCC [8,9]. New diagnostic, prognostic, and therapeutic approaches are therefore needed if the current weaknesses of CC clinical management are to be overcome and the life expectancy of these patients improved.

Molecular biomarkers have proved to be very useful in many cancer types, as they are objective, quantitative, and easy to reproduce and standardize among laboratories and hospitals [10,11,12,13,14]. They have significantly improved prediction of diagnosis, prognosis, and therapeutic response in breast [10], lung [15], and colorectal [16] cancers, and glioblastoma [17]. Thus, the use of molecular biomarkers supported by the understanding of CC molecular pathogenesis would enable a more accurate clinical handling of these patients [18]. In fact, the European Society of Medical Oncology has strongly recommended more research to identify molecular biomarkers in CC [2].

The pathogenic mechanisms of CC are not fully understood at present. Although it is widely recognized that persistent HR-HPV infection is the main cause of CC, since it is present in 99.7% of cases, additional molecular changes are necessary to immortalize and transform cervical epithelial cells [6,19]. Epigenetic alterations have been highlighted among these changes, because they could be useful tools for cancer screening, early detection, prognosis prediction, and therapy, given their reversible nature. While DNA methylation and histone modification have been widely studied in CC [6,18,20,21], the mechanisms and the role of micro-RNA (miRNA) deregulation in cervical carcinogenesis are still to be determined [19]. miRNAs are short non-coding RNAs that modulate gene expression by pairing with complementary nucleotide sequences, mainly of the 3′ untranslated region (3′UTR) of the target mRNA, leading predominantly to mRNA cleavage or protein translation repression [19,22,23]. Growing evidence reveals a profound deregulation of miRNA expression in cancer [23,24], including CC [19,22,25,26,27,28]. However, a very small overlap among those reports has been observed, probably due to the small sizes of the series analyzed, potential sample selection biases due to poor suitability of available material, and/or differences in study designs, populations, techniques, and data analyses [19,29,30,31]. All these issues have limited our wider understanding of the specific role of miRNAs in SCCC pathogenesis [19].

hsa-miR-877 is a human miRNA whose potential involvement in cancer has been reported in several tumor types [32,33,34,35]. In particular, the contribution of the mature miR-877-5p strand to cancer has been shown in non-small cell lung cancer [36] and glioblastoma [37], but mainly in tumors of the digestive tract, such as colorectal cancer [38,39], pancreatic ductal adenocarcinoma [40], hepatocellular carcinoma [41], and distal bile duct tumors [42]. Conversely, the involvement of the mature miR-877-3p strand in cancer has been demonstrated in tumors, such as glioma [43], and bladder [44], and gastric cancer [45]. Despite these studies, very few articles have so far shed any light on the miR-877 molecular mechanism in cancer, describing that miR-877 inhibited Toll-like receptor 4 (TLR4) expression in glioma [43], activated p16 expression in bladder cancer [44], and restored paclitaxel sensitivity in hepatocellular carcinoma [32]. Since TLR4 seems to be associated with inflammation response against HPV and initiation and progression of CC [46,47,48,49], p16 overexpression is a hallmark of cervical malignancies [3,4], and paclitaxel is a conventional chemotherapeutic agent for SCCC patients [2], we hypothesized that the miR-877-3p could play an important role in CC. Therefore, we aimed to explore the molecular and clinical involvement of miR-877-3p in cervical tumors.

## 2. Results

### 2.1. miR-877-3p is Overexpressed in Cervical Malignancies

**Figure 1:**
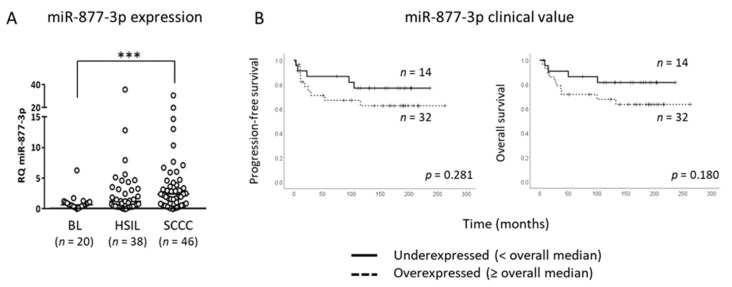
miR-877-3p in cervical tissues. (**A**) miR-877-3p expression was measured by quantitative PCR in a series of 104 lesions of the cervical epithelium with varying degrees of severity. (*** *p* < 0.001; RQ, relative quantification). (**B**) SCCC patients were stratified according to the level of miR-877-3p expression (overall median of the whole series of benign lesions (BLs), high-grade squamous intraepithelial lesions (HSILs), and squamous cell carcinomas of the cervix (SCCCs)). No significant associations were found between the miR-877-3p expression profile and progression-free or overall survival.

To explore the potential involvement of miR-877-3p in CC, its expression was measured by quantitative PCR (qRT-PCR) in a series of 46 SCCCs, 38 HSILs, and 20 BLs. We observed a very significant overexpression in tumors compared with BLs (*p* < 0.001) (Table 1 and Figure 1A). Furthermore, the clinical value of miR-877-3p expression was examined in SCCC. Several cut-off points were used to stratify patients according to their miR-877-3p levels, such as Youden’s index, the mean and median, and the 75th percentile of the series. Although no significant associations with survival were found, SCCC patients with higher miR-877-3p levels tended to have a worse prognosis than those with a lower level of expression, regardless of the cut-off value (Figure 1B). These findings suggested that miR-877-3p could have a biological role in SCCC.

### 2.2. miR-877-3p Silencing Is Not Critical for CC Cell Proliferation

**Figure 2:**
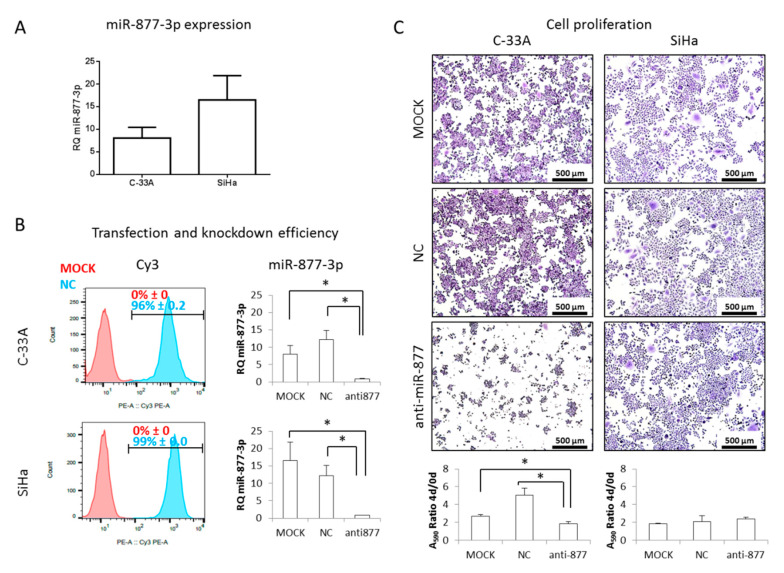
miR-877-3p inhibition in squamous cell carcinoma of the cervix (SCCC) cell lines. (**A**) miR-877-3p expression was measured by quantitative PCR in C-33A and SiHa cells. (**B**) SCCC cells were transfected with a Cy3-labelled negative control anti-miR (NC) and an anti-miR-877-3p. Transfection and knockdown efficiencies were checked by flow cytometry (left panel) and quantitative PCR (right panel), respectively. (**C**) Effect of miR-877-3p silencing on SCCC cell proliferation was analyzed after a 4-day transfection by staining cells with crystal violet and measuring the absorbance at 590 nm. Images were acquired at 50× magnification. (* *p* < 0.05; RQ, relative quantification).

To determine the biological role of miR-877-3p in SCCC, functional experiments were carried out in two cell lines. miR-877-3p levels were measured in C-33A and SiHa cells by qRT-PCR, and found to be expressed at higher levels (Figure 2A) than BLs. Therefore, a knockdown strategy was employed by using a Cy3-labelled scramble negative control anti-miR (NC) and an anti-miR-877-3p. Flow cytometry showed that transfection efficiency was greater than 96% in all cell lines (Figure 2B). Concomitantly, a time- and dose-dependent decrease in miR-877-3p levels was found by qRT-PCR upon anti-miR-877-3p transfection (data not shown), with more than 90% knockdown being achieved after 4 days (Figure 2B). This silencing affected cell proliferation in C-33A cells, but not in SiHa cells (Figure 2C). Effects of miR-877-3p inhibition were also analyzed in the HeLa cell line, which was derived from an HPV18-positive cervical adenocarcinoma, with clinical features nearly identical to those of SCCC [50]. Although HeLa cells had lower levels of miR-877-3p than did SCCC-derived cell lines (Figure S1A), the transfection efficiencies were very similar (Figure S1B), and no effect on HeLa cell proliferation was observed upon miR-877-3p inhibition (Figure S1C).

As no major differences were found in cell proliferation, with the exception of the C-33A cell line, we explored other processes relevant to cell survival, such as apoptosis induction, changes in the distribution of cell cycle phases, and p16 expression regulation, which has been reported to be activated by miR-877-3p in bladder cancer [44]. Again, no cell response was consistently modulated by miR-877-3p inhibition (Figure S2), indicating that miR-877-3p did not mainly affect cell survival pathways in CC.

### 2.3. miR-877-3p Silencing Impairs CC Cell Migration and Invasion

**Figure 3:**
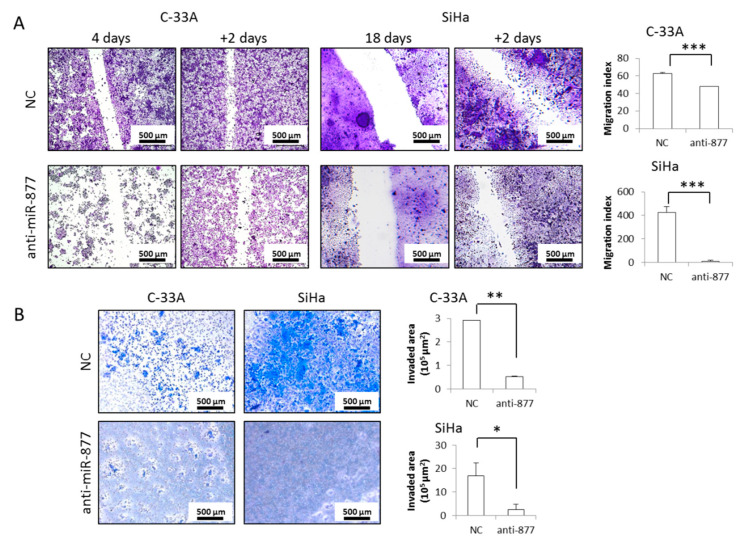
Effects of miR-877-3p inhibition on squamous cell carcinoma of the cervix cell migration and invasion. (**A**) C-33A and SiHa cells were transfected with the negative control anti-miR (NC) and the anti-miR-877-3p for 4 and 18 days, respectively, to reach nearly confluence, and cell migration ability was assessed 2 days later with a wound-healing assay. The migration index was calculated as the scratch width at first day minus that at +2 days. Images were acquired at 50× magnification. (**B**) C-33A and SiHa cells were transfected with the NC and the anti-miR-877-3p for 4 days. Cell invasion capacity was measured as the ability to penetrate a Matrigel^®^ layer over 3 days. Images were acquired at 50× magnification. (* *p* < 0.05; ** *p* < 0.01; *** *p* < 0.001).

We wondered whether miR-877-3p could be involved in later events in SCCC progression. After anti-miR-877-3p transfection, a 2-day wound-healing assay showed that miR-877-3p inhibition significantly impaired C-33A and SiHa cell migration (Figure 3A). We observed that cell invasion ability was almost completely abolished in both cell lines after miR-877-3p knockdown (Figure 3B). Very similar results regarding cell migration (Figure S1D) and invasion (Figure S1E) were observed in the HeLa cell line. These findings demonstrate that miR-877-3p is necessary for the acquisition of aggressive properties in CC, and highlight its potential as a therapeutic target in CC.

### 2.4. The ZNF177 Gene Is a Direct Target of miR-877-3p

**Figure 4:**
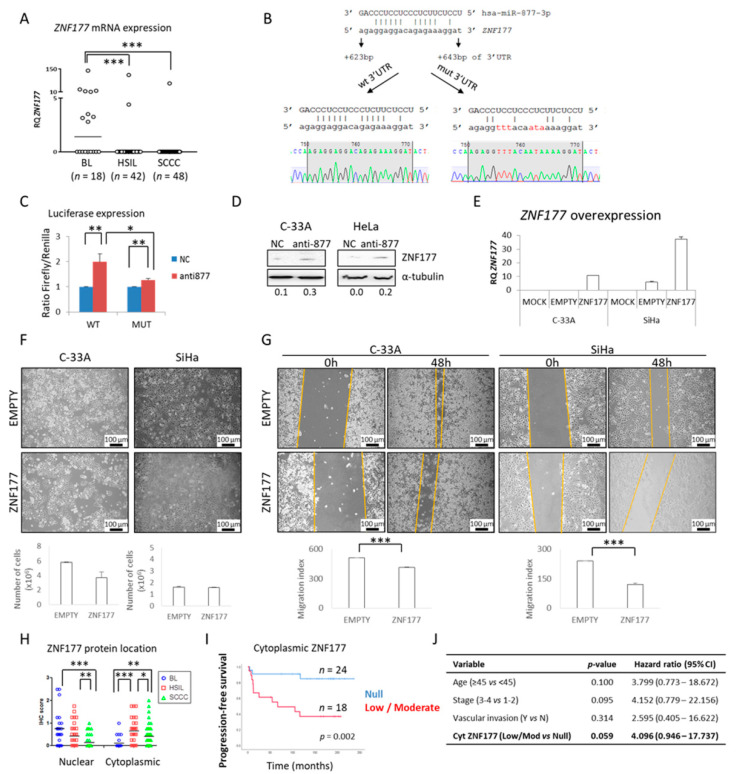
*ZNF177* expression in cervical cancer. (**A**) *ZNF177* mRNA levels were measured by quantitative PCR in a series of 108 cervical malignancies. Horizontal lines show the median of each series. (RQ, relative quantification). (**B**) A putative binding site of the miR-877-3p was found in the 3′ untranslated region (3′UTR) region of the *ZNF177* gene. Two 3′UTR regions were synthesized: one with the intact binding site (wild type, wt), and one with six mutated nucleotides (in red) from the binding site (mut), to weaken the potential binding to miR-877-3p. (**C**) 293T cells were transfected with both the pGL3-control luciferase reporter vector containing the firefly luciferase gene with either the wt or the mut 3′UTR of the *ZNF177* gene, and the *Renilla* firefly reporter vector, along with the negative control anti-miR (NC) or the anti-miR-877-3p. Luminescence was measured 3 days later. (**D**) ZNF177 protein levels were examined in CC cell lines upon miR-877-3p silencing, and α-tubulin was used as a loading control. Numbers indicate the ratio of ZNF177 to α-tubulin intensity assessed by densitometry. (**E**) Efficiency of *ZNF177* overexpression in C-33A and SiHa cells was measured by quantitative PCR in untransfected cells (MOCK), cells transfected with the empty vector pcDNA3.1 (EMPTY), and cells transfected with the pcDNA3.1 + ZNF177 construction (ZNF177). (**F**) 0.5 × 10^6^ untransfected and transfected cells with the empty vector and the vector + ZNF177 construction were seeded and allowed to grow for 72 h. They were then trypsinized and viable cells were counted by using the Trypan blue exclusion test. (**G**) Equally transfected cells were seeded and allowed to almost reach confluence. After 8 h fetal bovine serum (FBS)-starvation, 3 scratches were performed on the cell monolayer, and cells were allowed to migrate to heal the wound for 48 h in the presence of 5% FBS. Migration index was calculated as the scratch width at 0 h minus that at 48 h. All images were acquired at 100× magnification. (**H**) Nuclear and cytoplasmic ZNF177 protein levels were evaluated by immunohistochemistry in 98 cervical malignancies (23 benign lesions (BLs), 33 high-grade squamous intraepithelial lesions (HSILs), and 42 squamous cell carcinomas of the cervix (SCCCs)). (**I**) Association between cytoplasmic ZNF177 expression levels and progression-free survival in SCCC patients. (**J**) Multivariate analysis showing almost significant independence of cytoplasmic ZNF177 expression as a potential prognostic biomarker in SCCC (*p* = 0.059). (CI, confidence interval) (* *p* < 0.05; ** *p* < 0.01; *** *p* < 0.001).

Two strategies were used to gain insight into the miR-877-3p molecular mechanism. First, in silico target prediction was analyzed using the TargetScan v7.1 database (http://www.targetscan.org/) [51] (accessed on April 2016). Of all the predicted targets, the zinc finger protein 177 (*ZNF177*) gene was selected because it has been described as being epigenetically silenced in other tumor types [52,53,54], including gynecological cancers [55,56]. Therefore, *ZNF177* expression was measured by qRT-PCR in our series of cervical tissues. Interestingly, a higher level of *ZNF177* was found in BLs than in cervical tumors, HSILs, and SCCCs (Figure 4A), suggesting that there is an inverse relationship with miR-877-3p expression (Table 1 and Figure 1A). Notably, we identified a potential binding site of miR-877-3p at 623–643 bp of the *ZNF177* gene 3′UTR region. To demonstrate their functional interaction, two 3′UTR regions of the *ZNF177* gene were cloned into the pGL3-control luciferase reporter vector: one with an intact potential binding site (wild type, wt 3′UTR), and another with six nucleotides mutated at the binding site (mut 3′UTR) to weaken the binding (Figure 4B). After transfection into 293T cells, we found that the anti-miR-877-3p was able to significantly induce luciferase expression compared with the NC, and that the increase was more pronounced in the wt 3′UTR than in the mut 3′UTR (Figure 4C). Accordingly, upregulation of the ZNF177 protein level was observed by western blot in CC cell lines upon miR-877-3p knockdown (Figure 4D), indicating that *ZNF177* expression is negatively regulated by miR-877-3p. Furthermore, *ZNF177* overexpression (Figure 4E) did not affect SCCC cell proliferation (Figure 4F), but significantly impaired cell migration ability in both C-33A and SiHa cell lines (Figure 4G), as miR-877-3p inhibition did (Figure 3A). Collectively these findings suggest a functional link between miR-877-3p and ZNF177.

Additionally, ZNF177 expression was explored in tissues from cervical malignancies. Although this protein has been little researched, it is predicted to be a transcription factor due to its zinc-finger structure. First, we examined its nuclear expression, and found that it dropped significantly as the severity of the lesion increased from BL to SCCC (Figure 4H, Figure S3, and Table 2). Importantly, we noticed that cytoplasmic ZNF177 levels were significantly higher in cervical tumors than in BLs. This observation suggested that ZNF177 expression and subcellular location could be used as diagnostic biomarkers to distinguish HSIL from SCCC. In fact, as shown in Figure S4, there was a significant correlation between nuclear but not cytoplasmic ZNF177 levels and the type of cervical lesion (R^2^ = 0.9859, *p =* 0.01; and R^2^ = 0.0453, *p* = 0.667, respectively). Intriguingly, SCCC patients with some cytoplasmic ZNF177 expression (although at a low level) had a very significantly worse prognosis than those with no cytoplasmic ZNF177 (*p* = 0.002) (Figure 4I) and tended to have worse overall survival too, although statistical significance was not reached (*p =* 0.079) (Figure S5). This finding highlighted the value of cytoplasmic ZNF177 expression as a potential prognostic biomarker in SCCC, representing a four-fold increase in the risk of relapse almost regardless of other clinical characteristics relevant to SCCC progression (*p* = 0.059), such as age, stage and vascular invasion (Figure 4J). Furthermore, a preliminary estimation of the accuracy of ZNF177 subcellular location to predict prognosis and diagnosis in CC was performed. Figure S6 shows good sensitivity and specificity, with area under the curve of 0.767 and 0.838 for prognosis and diagnosis, respectively (*p* ≤ 0.001). While aware of the low number of cases studied, these results indicate that cytoplasmic ZNF177 expression and subcellular location could be used to predict diagnosis and prognosis in cervical malignancies.

### 2.5. miR-877-3p Is Involved in Regulating Cytoskeletal Protein Folding

**Figure 5:**
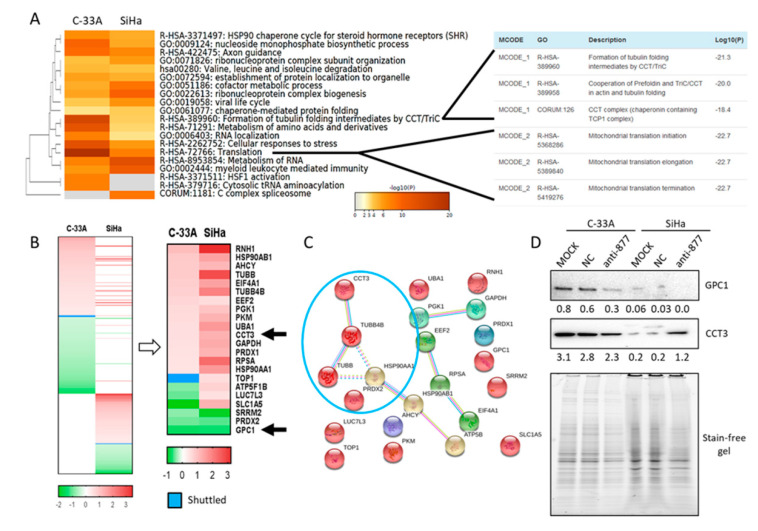
Integration of proteins significantly deregulated by miR-877-3p in both squamous cell carcinoma of the cervix (SCCC) cell lines. (**A**) Proteins simultaneously altered by miR-877-3p in C-33A and SiHa cell lines were functionally clustered by the Metascape tool. Details are provided for the most significant terms: formation of tubulin folding intermediates by CCT/TRiC complex and protein translation. (**B**) Heat-maps showing all proteins that were significantly altered by anti-miR-877-3p in each cell line (left panel), and the 22 commonly altered proteins in both cell lines (right panel). Black arrows highlight CCT3 and GPC1 proteins, which were selected for further validation. Scale bars represent log_2_-fold change. Blue color indicates proteins that shuttled from the nucleus to the cytoplasm in C-33A, or from the cytoplasm to the nucleus in SiHa. (**C**) Network of the 22 proteins commonly deregulated by the anti-miR-877-3p in both C-33A and SiHa cell lines. The blue circle highlights the strong link between chaperones and cytoskeletal proteins. (**D**) Western blots showing decreased and increased levels of GPC1 and CCT3 protein levels, respectively, in SCCC cells upon miR-877-3p inhibition. An image of the whole stain-free gel was used to check protein loading; no antibody was employed for this purpose because most of them detect cytoskeletal proteins or metabolic enzymes like GAPDH, which appeared here as a differentially expressed protein. Numbers indicate the intensity of each band relative to that of the loaded protein, as measured by densitometry.

In parallel, we used a second strategy to understand the mechanism of action of miR-877-3p, based on the exhaustive characterization of the proteostatic impairment induced by miR-877-3p knockdown. Given the importance of ZNF177 subcellular location, cytoplasmic, and nuclear proteomes of NC-transfected and anti-miR-877-3p-transfected SCCC cells were separately extracted and compared by liquid chromatography-tandem mass spectrometry (LC–MS/MS) (Table S1). Since no striking changes in protein shuttling between subcellular components were found, data from both fractions were integrated as whole-cell proteomes. Thus, 180 and 114 human proteins were found to be significantly altered upon miR-877-3p knockdown in C-33A and SiHa cell lines, respectively (*p* < 0.05) (Tables S2 and S3). Notably, no HPV16 protein was detected in SiHa cells. Overall, protein upregulation was a more frequent event than protein downregulation (92 and 76 upregulated vs. 78 and 38 downregulated proteins in C-33A and SiHa, respectively). All differentially expressed proteins in each cell line were clustered into two major biological groups, one involving protein synthesis, and the other linking chaperones and cytoskeletal proteins. These functions were retrieved using several bioinformatic tools, such as STRING (Figure S7), Ingenuity Pathway Analysis (Tables S4 and S5, and Figure S8), and Metascape (Figure 5A). When data from the two cell lines were combined, the most significant clusters were protein translation and formation of tubulin folding intermediates by the chaperonin-containing T-complex protein 1 (CCT) complex (Figure 5A). To identify which proteins were involved in these processes, the 22 proteins that were simultaneously altered by miR-877-3p in both cell lines (Figure 5B) were integrated in a network, which highlighted a strong functional interaction between CCT3 and HSP90AA1 chaperones, and TUBB4B and TUBB cytoskeletal proteins (Figure 5C). Finally, two proteins were selected to validate these results: GPC1, which displayed the greatest change, and CCT3, which seemed to play a central role in miR-877-3p pathway. In accordance with the data-mining analysis, GPC1 and CCT3 protein expression decreased and increased, respectively, upon miR-877-3p silencing, especially in SiHa cells (Figure 5D). These findings confirm that miR-877-3p regulates cytoskeletal protein folding, which would explain the impairment of cell migration and invasion observed when it is knocked down.

### 2.6. miR-877-3p Knockdown Synergizes with Paclitaxel

**Figure 6:**
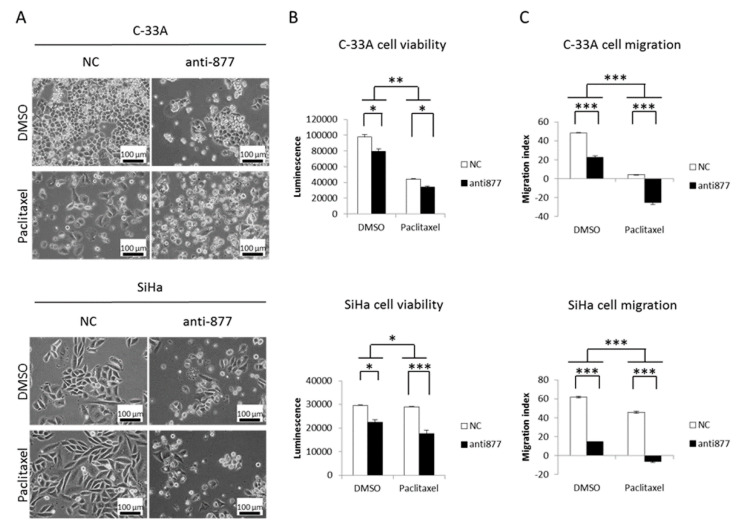
Simultaneous miR-877-3p inhibition and paclitaxel treatment in squamous cell carcinoma of the cervix cell lines. Anti-miR-877-3p transfection and sub-IC_50_ paclitaxel treatment were performed in C-33A and SiHa cells. Effects on cell morphology, viability, and migration were monitored for 24, 48 and 72 h. (**A**) Images were acquired at 200× magnification after 72 h of miR-877-3p silencing and paclitaxel treatment. (**B**) C-33A and SiHa cell viabilities were measured after 72 h of double-targeting as the intracellular ATP content. (**C**) C-33A and SiHa cell migration was examined 48 h after double-targeting. The migration index was calculated as the scratch width at time 0 minus that at 48 h. (* *p* < 0.05; ** *p* < 0.01; *** *p* < 0.001).

In view of the disruption of cytoskeletal protein folding driven by the miR-877-3p, we examined whether these effects could be enhanced by paclitaxel, a microtubule-stabilizing drug commonly administered to SCCC patients [2]. To this end, first, half maximal inhibitory concentration (IC_50_) values of paclitaxel were calculated at 72 h. As seen in Figure S9, paclitaxel was very effective in all CC cell lines in the low nanomolar range. SCCC cells were then simultaneously subjected to miR-877-3p silencing and sub-IC_50_ paclitaxel treatment for 24, 48, and 72 h. We observed a noticeable change in cell morphology (Figure 6A), and a significant drop in cell viability (Figure 6B) and especially in cell migration (Figure 6C) in SCCC cells. Similar though smaller magnitude results were observed in the HeLa cell line (Figure S10). Importantly, all these effects were significantly enhanced relative to single miR-877-3p knockdown or paclitaxel administration. The observed inhibitory effect of the combination was greater than the expected one (Table 3), indicating that the double-targeting of cytoskeletal proteins had a synergistic effect on CC cell viability and migration.

## 3. Discussion

Clinical management of cervical cancer is still limited because of the lack of molecular biomarkers that could help clinicians diagnose, provide a prognosis and propose new targeted therapies [2,3,4,5], especially for SCCC, which represents 70–80% of all CC cases [2]. A better understanding of the molecular mechanisms that underlie SCCC development would allow us to identify the biomarkers we need to improve the dismal expectancies of these patients [2,18,29]. Among the molecular aberrations that arise during SCCC pathogenesis, miRNAs have attracted increasing attention, because they affect the expression of tumor-suppressor genes or oncogenes, which in turn, alter multiple biological processes, such as cell maturation, differentiation, proliferation, cell cycle, migration, invasion, autophagy, apoptosis, adhesion, and metastasis [29]. Aberrantly expressed miRNAs in CC patients compared with healthy women have been extensively reported [28,57], and some of them have been proposed as biomarkers of potential use in CC diagnosis [25,26,27,58], prognosis [19,22,29,30,59] or response to radiotherapy [60,61,62] and chemotherapeutic agents, such as cisplatin [63,64], and paclitaxel [65]. Here, we focused on miR-877 because of its association with processes relevant to SCCC, such as p16 activation [3,4], restoration of paclitaxel sensitivity [2], and TLR4 inhibition [46,47,48,49], in bladder cancer [44], hepatocellular carcinoma [32], and glioma [43], respectively. Our results demonstrate that miR-877-3p is overexpressed in tumoral samples relative to BLs and, for the first time, provide evidence of the contribution of miR-877-3p in SCCC progression. Very recently, it has been described that the miR-877-5p strand may play an anti-tumor role in CC [66], because it is downregulated in CC tissues, and its overexpression decreased CC cell proliferation and invasion [67]. In spite of the apparent controversy between this recent report and our results, it is important to note that the mature 5p and 3p strands of the same miRNA can participate in different cell signaling pathways, and then have distinct effects [68,69].

Although miR-877-3p silencing did not seem to affect CC cell proliferation, apoptosis induction, or cell cycle deregulation, we observed that miR-877-3p had a major role in CC cell migration and invasion, processes associated with the later stages of cancer progression. Similarly, miR-877-3p did not influence p16 expression in SCCC, unlike what has been observed in bladder cancer [44]. Since p16 is an essential cell-cycle regulator in cervical carcinogenesis [3,4,70,71], the lack of association between miR-877-3p expression and p16 levels strengthened the idea that miR-877-3p was not involved in the early stages of SCCC development. Likewise, miR-20a [72,73] and miR-218 [74] were found to promote CC cell migration and invasion without affecting cell viability. In fact, although the evidence points to a role for miRNAs at every stage of CC initiation and development [22], many miRNAs have very recently been linked to aggressiveness in CC [75,76,77,78,79,80,81,82,83], although their complex molecular pathways have not been fully elucidated. Here, we have used two strategies to explore the mechanism of action of miR-877-3p in SCCC: in silico-predicted target examination and comprehensive identification of proteins whose expression changed in vitro upon miR-877-3p inhibition. In spite of the large amount of data these approaches can produce, it is important to note that they also have limitations: on the one hand, in silico prediction is based on the complementarity between miRNA and target 3′UTR sequences, and therefore requires careful validation. On the other hand, for technical reasons, only the most abundant proteins can be explored by our LC–MS/MS approach. Consequently, miR-877-3p targets other than those reported in this study might also be active in its molecular pathway. For instance, no HPV16 protein was detected in our experiments in miR-877-3p-silenced SiHa cells, probably because of its lower relative abundance compared with human proteins. However, a link between miR-877-3p and HPV16 cannot be ruled out.

Some reports have shed light on miRNA machinery by identifying the particular protein targets responsible for cancer-cell aggressiveness. For example, the tumor-suppressor miR-218 targeted LAMB3, a laminin-332 component of the basement membrane which influences cell adhesion, migration and invasion in SCCC [74] through cytoskeleton organization [84]. Then, disassembly of cytoskeletal structures, which is crucial for gaining cell motility and initiating metastasis [85], can also be controlled by miRNAs. Consistent with this, we show that miR-877-3p promoted CC cell migration and invasion by modulating the folding of cytoskeletal proteins, mainly through the CCT complex, also known as the TCP-1 ring complex (TRiC). CCT/TRiC is a chaperone complex, comprising eight subunits (CCT1-8), which is required for the folding of newly synthesized actin and tubulin, and is thus linked to all cellular processes involving microtubules and actin filaments, such as cell division, migration, and invasion, which are major drivers of cancer progression [86,87,88]. The contribution of CCT/TRiC to cancer has not received much attention, but evidence of it is now emerging, since it also mediates the folding and function of client proteins related to oncogenesis, such as the Von Hippel-Lindau and p53 tumor-suppressor proteins, the telomerase cofactor TCAB1 and STAT3 oncoproteins, and several cell cycle regulators [88]. The disrupted folding activity of CCT/TRiC can result in altered proteostasis, leading to loss or toxic gain of function of the misfolded substrates. It has been suggested that each CCT subunit has specific substrates and, therefore, different functions [88,89]. For instance, CCT8 regulates cell proliferation, migration and invasion in several tumors [90,91,92,93]; CCT2 inhibition reduces cell migration in tubulin-binding agent-resistant tumors [88,94,95,96]; and CCT3 supports cell proliferation in hepatocellular carcinoma [97]. Based on our findings, the anti-miR-877-3p increased CCT3 expression in SCCC, leading to an anomalous folding of actin and tubulin, which impaired both cell migration and invasion. Accordingly, although only one CCT subunit, CCT6A, has been found among the miR-877-3p in silico predicted targets in the TargetScan database (most recently accessed in March, 2021), we did find among them some cytoskeletal proteins, such as ACTC1 (actin, alpha cardiac muscle 1), ACTRT3 (actin-related protein T3), PHACTR2 (phosphatase and actin regulator 2), PHACTR3 (phosphatase and actin regulator 3), ACTN1 (alpha-actinin-1), LAMC3 (laminin subunit gamma-3), and TUBB6 (tubulin beta-6 chain). Since microtubules are already the target of therapies such as paclitaxel, it has been suggested that inhibiting CCT-tubulin interactions, and then tubulin folding, may enable new therapeutic strategies for treating tumors that have developed resistance to paclitaxel [86]. In fact, we demonstrate that miR-877-3p silencing synergistically enhances the effects on CC cell viability and, above all, cell migration induced by paclitaxel, one of the most common drug for SCCC patients [2]. It is well established that paclitaxel commonly induces mitotic arrest [98,99], so low doses were used here to avoid massive cell detachment and loss of cell monolayer in our attempt to measure migration ability, as previously reported [100,101]. Even at sub-IC_50_ doses of paclitaxel, the reduction of CC cell migration is synergistically potentiated by miR-877-3p depletion. Therefore, our results highlight the relevance of cytoskeleton-targeting as an effective therapeutic strategy in CC, and suggest that knocking down miR-877-3p could improve therapeutic response and reduce potential toxicity and side effects by allowing a lower paclitaxel dose to be administered.

Almost half the patients who are diagnosed with locally advanced CC have an unfavorable response to conventional treatments based on cisplatin and paclitaxel [1,2,102]. A growing body of evidence has shown that miRNA manipulation may be beneficial for CC treatment. Thus, the therapeutic development of miRNA mimics (to recover tumor-suppressor miRNA levels) or inhibitors (to repress onco-miR expression) may facilitate novel therapies and improve CC prognosis [22,25], as did miR-26a, miR-31, and miR-34a delivery in liver, breast, and advanced solid tumors, respectively [26,30]. Regarding CC, in vivo miR-143 or miR-34 supplementation, as well as miR-21 inhibition, reduce metastatic potential, and so have been proposed as useful therapeutic strategies [25]. In the present study, we also propose miR-877-3p as a potential therapeutic target in CC, since we have demonstrated that its inhibition diminishes cell migration and invasion and improves response to paclitaxel in vitro. Further research is required to test the effects of miR-877-3p silencing alone and combined with paclitaxel in in vivo CC models.

Since miRNA deregulation plays a key role in CC malignant transformation, there has been increasing interest not only in miRNAs per se, but also in their targets, since both can be exploited in the clinical setting [22]. We have experimentally verified that the *ZNF177* gene is directly targeted and repressed by miR-877-3p, and that is functionally involved in the effects on cell migration driven by miR-877-3p. As an in silico predicted target of miR-877-3p, *ZNF177* has been little investigated. A few cancer types are known to exhibit epigenetic inhibition of *ZNF177* expression. In particular, a higher frequency of aberrant DNA methylation of its promoter has been reported in endometrial [55], lung [52], hepatocellular [53], and gastric [54] cancers compared with their non-tumoral counterparts. Indeed, *ZNF177* is part of the signature of hypermethylated genes of diagnostic value in early stages of lung [52] and breast cancer [56]. Similarly, we have established the clinical importance of assessing ZNF177 expression and subcellular location in cervical malignancies, whose clinical management urgently needs molecular biomarkers for an accurate prognosis and diagnosis [2,5], differentiating HSIL and SCCC cases [8,9]. Importantly, ZNF177 expression and subcellular location effectively distinguish HSIL from SCCC. This is the first report of a molecular biomarker that could be useful in cervical tumor diagnosis, which otherwise currently depends on the subjective assessment of tissue morphology [2,3,4,5]. We also highlight the importance of ZNF177 subcellular location in predicting poor prognosis in SCCC. Although the borderline statistical significance did not establish cytoplasmic ZNF177 as an independent prognostic biomarker in our series (*p* = 0.059), further research in a larger cohort of patients might confirm its independent value. The vast majority of molecular biomarkers currently used in clinical practice are based on the aberrant levels of some proteins (mostly presence/absence, such as hormone receptor and HER2 in breast cancer [10]), rather than on their subcellular location. However, regardless of expression levels, if a protein is not located where it should exert its activity, it may not function properly. Therefore, changes in protein location could be also considered as molecular alterations, and thus, potential biomarkers. Indeed, we [103], and others [104,105,106,107,108,109], have reported the likely clinical relevance of the subcellular location of some proteins in several tumors. Similarly, we emphasize here for the first time the relevance of the subcellular location of ZNF177 as a potential diagnostic and prognostic biomarker in SCCC. Nevertheless, the likely value of ZNF177 as biomarker needs to be confirmed in larger cohorts of patients.

## 4. Materials and Methods

### 4.1. Patient Samples

A series of 117 formalin-fixed, paraffin-embedded (FFPE) samples from women diagnosed with SCCC (*n* = 52), HSIL (*n* = 42) and BL (*n* = 23), with International Classification for Oncology codes 8070/3, 8077/2, and 8052/0, respectively, was analyzed. All patients were diagnosed between 1995 and 2015 in the Pathology Departments of the Complejo Hospitalario de Navarra (Pamplona, Spain), Hospital Universitari de Bellvitge, (L’Hospitalet de Llobregat, Spain), Complejo Asistencial Universitario (León, Spain), and Hospital Universitari Arnau de Vilanova (Lleida, Spain). SCCC patients’ demographic, pathological, and clinical characteristics are summarized in Table S6. No clinical follow-up was available for HSIL or BL patients, since they were all successfully cured by surgery. All tumors were surgically removed and staged according to their size, histological grade, and lymph node involvement. All cases were ensured to harbor at least 70% tumor cells, and none of the patients had received radiotherapy or chemotherapy before surgery. The study was approved by the Navarre Ethics Committee for Clinical Research (PI_2018/75) on 30 September, 2019, procedures were in accordance with the Declaration of Helsinki of 1975 and revised in 2013, and biopsies were obtained in accordance with current Spanish legislation regarding informed consent. All samples were double-coded by clinicians and researchers to ensure anonymity.

### 4.2. Cell Lines

Three human CC cell lines were used in this study: two SCCC-derived cell lines (C-33A and SiHa, HPV-negative and HPV16-positive SCCCs, respectively), and a cell line derived from an HPV18-positive cervical adenocarcinoma (HeLa). Additionally, 293T cells were used for dual-luciferase reporter assays. All cell lines were purchased from the American Type Cell Collection (Rockville, MD, USA). They were all grown in DMEM supplemented with 10% fetal bovine serum (FBS) and 1% penicillin/streptomycin (all from Thermo Fisher Scientific, Waltham, MA, USA), at 37 °C in a humidified atmosphere with 5% CO_2_. Experiments were performed with *Mycoplasma*-free and recently authenticated cell lines at low passage.

### 4.3. RNA Extraction and Quantitative Reverse-Transcription Polymerase Chain Reaction (qRT-PCR)

To determine miR-877-3p and *ZNF177* mRNA levels in cervical samples, the RNA fraction was first isolated from our series of FFPE samples and CC cell lines using the RecoverAll^TM^ total nucleic acid isolation kit (Thermo Fisher Scientific, Waltham, MA, USA) following the manufacturer’s instructions. Briefly, 15-µm sections were deparaffinized with xylene and dried with 100% ethanol in a vacuum for 30 min at room temperature. Samples were protease-digested for 15 min at 50 °C followed by 15 min at 80 °C. RNA was precipitated with an isolation additive and ethanol mixture, washed, DNase-treated for 30 min at room temperature, and washed again using filter columns in the kit. Finally, RNA was eluted from the column with 30 µL of RNase-free water, and concentration and quality were assessed using a NanoDrop spectrophotometer (Thermo Fisher Scientific, Waltham, MA, USA).

Next, qRT-PCR was performed as follows. For miR-877-3p expression, 100 ng of total RNA per each miRNA to be analyzed were retrotranscribed using the TaqMan^®^ microRNA reverse transcription kit and specific TaqMan^®^ primers for each miRNA (hsa-miR-877-3p: 241029_mat; and endogenous RNU48: 001006) at 16 °C for 30 min, 42 °C for 30 min and 85 °C for 5 min. One µL of the resulting cDNA was mixed with the TaqMan^®^, No AmpErase^®^ UNG universal PCR master mix and specific TaqMan^®^ probes for each miRNA (all reagents from Thermo Fisher Scientific, Waltham, MA, USA). For *ZNF177* mRNA expression, 200 ng of total RNA were retrotranscribed using the PrimeScript^TM^ RT reagent kit (TaKaRa, Otsu, Japan) at 37 °C for 15 min and 85 °C for 5 s. One µL of cDNA was mixed with specific TaqMan^®^ probes (*ZNF177*: Hs.PT.58.25734794; and the endogenous *GAPDH*: Hs.PT.39a.22214836, both from Integrated DNA Technologies, Coralville, IA, USA), and the buffer included in the Premix Ex Taq^TM^ kit (TaKaRa, Otsu, Japan). All PCR amplifications were performed in triplicate using the Quant Studio 12K Flex (Thermo Fisher Scientific, Waltham, MA, USA) under thermal cycler conditions of: (i) 95 °C for 10 min and 40 cycles at 95 °C for 15 s and 60 °C for 1 min, to amplify miRNAs; and (ii) 95 °C for 30 s and 40 cycles at 95 °C for 5 s and 60 °C for 34 s, for mRNAs. The cycle threshold (Ct) values were calculated using Quant Studio software (Thermo Fisher Scientific, Waltham, MA, USA), and the relative quantification (RQ) was calculated by the ΔCt method (RQ = 2^−ΔCt^), using RNU48 or *GAPDH* as the endogenous miRNA or gene control, respectively.

### 4.4. miR-877-3p Silencing in CC Cell Lines

To study the functional role of miR-877-3p in CC, C-33A, SiHa and HeLa cell lines were transfected with an anti-miR-877-3p (Cat# AM17000, ID AM13557) and a Cy3-labelled anti-miR scramble as a negative control (NC) (Cat# AM17011), which did not target any known human miRNA (both from Thermo Fisher Scientific, Waltham, MA, USA), at several concentrations and times. Briefly, 1 × 10^5^ cells/well were plated in six-well plates and allowed to attach overnight. Next day, anti-miRs were diluted in 200 µL of DMEM with 2 µL of the INTERFERin^®^ transfection agent (Polyplus-transfection SA, Illkirch, France), incubated for 10 min at room temperature and added to the cells. Transfection efficiency was checked by flow cytometry: at each time, non-transfected (MOCK) and NC-transfected cells were harvested, washed twice with phosphate-buffered saline (PBS), and resuspended in 200 µL of PBS. The percentage of Cy3-positive cells was then determined by acquiring 10,000 cells in a FACS Canto flow cytometer (Beckton Dickinson, BD, Franklin Lakes, NJ, USA) with the FACS DIVA (BD, Franklin Lakes, NJ, USA) and the FlowJo programs (LLC, Ashland, OR, USA). Furthermore, miR-877-3p levels were measured by qRT-PCR, as described above, in MOCK, NC-transfected and anti-miR-877-3p-transfected cells to check knockdown efficiency.

### 4.5. Effects of miR-877-3p Inhibition on CC Cell Survival

To test the functional consequences of miR-877-3p knockdown on CC cell survival, C-33A, SiHa and HeLa cell lines were transfected with NC and anti-miR-877-3p for 4 days, and several vital characteristics were measured. First, cell proliferation was interrogated by fixing and staining cells with a paraformaldehyde-containing crystal violet solution (Sigma-Aldrich, St Louis, MO, USA). Images were acquired with a Leica DM4000B microscope (Leica, Wetzlar, Germany) and the NIS Elements program (Nikon Instruments, Amsterdam, Netherlands) at 50× magnification. Absorbance at 590 nm was measured with an Epoch plate reader (BioTek, Winooski, VT, USA). Second, apoptosis induction was examined by flow cytometry using the APC-Annexin V binding assay. Briefly, MOCK, NC-transfected and anti-miR-877-3p-transfected cells were harvested, washed twice with PBS, and resuspended in 200 µL of Annexin V-binding buffer with 2 µL APC-Annexin V (BioLegend Way, San Diego, CA, USA) and 0.5 µL of DAPI. Data were recorded on a FACS Canto flow cytometer, as described above. Finally, cell distributions during each cell cycle phase were also evaluated by flow cytometry. Similarly, MOCK, NC-transfected and anti-miR-877-3p-transfected cells were harvested, washed twice with PBS, fixed with ice-cold 70% ethanol for at least 1 h at 4 °C, and resuspended in 200 µL of PBS with 10 µL of propidium iodide and 1 µL of RNase A for 30 min at room temperature in darkness, and analyzed using a FACS Canto flow cytometer, as described above.

### 4.6. Cell Migration

In order to test the effects of miR-877-3p knockdown on CC cell migration, C-33A, SiHa, and HeLa cells were seeded and transfected with NC and anti-miR-877-3p until confluence was almost reached. Cells were then FBS-starved for 8 h, and three scratches were made on the cell monolayer with a 10-µL pipette tip. Some cells were fixed and stained with crystal violet, while others were maintained for two more days in a 5% FBS containing medium, and then fixed and stained. Finally, 10 pictures were taken with a Leica DM4000B microscope (Leica, Wetzlar, Germany) at 50× magnification, and the scratch width was measured to calculate the migration index as the scratch width at 0 days minus that at 2 days, with the NIS Elements program (Nikon Instruments, Amsterdam, Netherlands), from more than 10 measurements taken from each picture, as previously described [110].

### 4.7. Cell Invasion

The effects of miR-877-3p silencing on CC cell invasion were also determined. To do this, C-33A, SiHa and HeLa cells were transfected with NC and anti-miR-877-3p, as described above, for 4 days. Cells were FBS-starved overnight, trypsinized and 1.25 × 10^5^ cells were re-seeded in FBS-free DMEM on a 40-µL Matrigel^®^ layer (BD, Franklin Lakes, NJ, USA), which had been previously allowed to gel for 15–30 min at 37 °C in Transwell^®^ inserts with an 8-µm-pore membrane (Sarstedt, Nümbrecht, Germany). Inserts were placed in wells with 10% FBS-containing medium, and maintained for three more days. Finally, the gel layer was removed with a cotton-tipped swab, and invading cells that had reached the membrane were fixed and stained with a paraformaldehyde-containing crystal violet solution. Images were captured under a Leica DMi1 microscope with the Leica Application Suite v4.12 program (Leica, Wetzlar, Germany) at 50× magnification. The area occupied by invading cells was measured with the NIS Elements program (Nikon Instruments, Amsterdam, Netherlands).

### 4.8. Dual-Luciferase Reporter Assay

A dual-luciferase reporter assay was carried out to test whether the expression of the in silico predicted target *ZNF177* was actually regulated by the miR-877-3p. To this end, the wild type (wt) 3′UTR region of the *ZNF177* gene was obtained from Thermo Fisher Scientific (Waltham, MA, USA). Similarly, a mutated (mut) 3′UTR region was also purchased, in which six nucleotides strategically located in the putative miR-877-3p-binding site were modified from the wt sequence in order to weaken the potential binding. The wt and mut 3′UTR regions were both cloned into the pGL3-control luciferase reporter vector, which contained the firefly luciferase gene without the 3′UTR region (kindly provided by Dr. Rubén Hernández-Alcoceba, Gene Therapy Program, Center for Applied Medical Research, Pamplona, Spain). To achieve this, plasmids were digested with the XbaI restriction enzyme and ends from the pGL3-control vector were dephosphorylated with FastAP thermosensitive alkaline phosphatase for 40 min at 37 °C and for 20 min at 80 °C to inactivate the enzyme. Digested plasmid and inserts were incubated with 1 µL of the T4 ligase for 20 min at room temperature. TOP10 Chemically Competent *Escherichia coli* bacteria were heat-shock-transformed with 4 µL of the ligation reaction (all reagents from Thermo Fisher Scientific, Waltham, MA, USA). Cloning was confirmed by XbaI-digestion and sequencing using the 5′-CGTCGCCAGTCAAGTAACAA-3′ primer. Finally, 293T cells were cotransfected with the cloned firefly luciferase + ZNF177-3′UTR insert, the *Renilla* luciferase reporter plasmid (also kindly provided by Dr. Rubén Hernández-Alcoceba, Gene Therapy Program, Center for Applied Medical Research, Pamplona, Spain), and the NC or the anti-miR-877-3p, as explained above. After 48 h, firefly and *Renilla* luminescence were measured using the DUAL-Glo^®^ luciferase assay system kit (Promega, Madison, WI, USA) in an Epoch plate reader (BioTek, Winooski, VT, USA), following the manufacturer’s instructions, and the ratio of firefly to *Renilla* luciferase expression was calculated.

### 4.9. ZNF177 Overexpression

The coding region of the *ZNF177* gene (Thermo Scientific, Waltham, MA, USA) was cloned into the pcDNA3.1 plasmid (Addgene, Watertown, MA, USA) using BamHI and NotI restriction enzymes (Thermo Scientific, Waltham, MA, USA) and *E. coli* XL-1 blue bacteria. Insert ligation was checked by digestion with BamHI and NotI restriction enzymes, and by sequencing. Due to the large size of the amplicon (1967 bp), two sequencing primers were used: 5′-TGTGATGCTGGAGAACTTTAGG-3′, and 5′-GGCCGCCTATTTGTCATCATC-3′. After checking ligation, SCCC cell lines were transfected with the empty vector and the vector + *ZNF177* construct using FuGene6 (Promega, Madison, WI, USA). Transfected C-33A and SiHa cells were selected with 500 µM and 1000 µM of G418 (Invivogen, Toulouse, France), respectively, for 13 days. Transfection efficiency was then checked by measuring *ZNF177* mRNA levels by qRT-PCR, as described above. Effects of *ZNF177* overexpression on SCCC cell proliferation and migration were evaluated at 72 h and 48 h, respectively, as explained above.

### 4.10. Subcellular Fractionation

Cells were lysed with a PBS-based buffer containing 1% NP-40 (Thermo Scientific, Waltham, MA, USA) and protease inhibitors (Roche, Basel, Switzerland), scraped, and centrifuged at 13,000 rpm for 1 min at 4 °C. Cytoplasmic proteins in the supernatant were collected and precipitated with ice-cold acetone, while pellets were resuspended in 50 µL of lysis buffer (7M urea, 2M thiourea, and 50 mM DTT), incubated on ice for 30 min and sonicated for two cycles of 20 s each. Finally, samples were centrifuged at 20,000× *g* for 20 min at 15 °C, and nuclear proteins were collected from the supernatant. Protein concentration was measured in the supernatants with the Bradford assay kit (Bio-Rad, Hercules, CA, USA). Detection of GAPDH protein preferentially in the cytosolic fraction, in addition to major histone H3 location in the nuclear fraction, indicated the efficiency of the enrichment procedure (Figure S11).

### 4.11. Label-Free Liquid Chromatography–Tandem Mass Spectrometry (LC–MS/MS)

The discovery phase (shotgun proteomics) was performed in two subcellular fractions (nucleus and cytoplasm) from two SCCC cell lines (C-33A and SiHa) with three biological replicates for each experimental condition: MOCK, NC-transfected and anti-miR-877-3p-transfected cells (36 samples in total). The size of the groups meant that the biological variation could be evened out. Protein extracts for each sample were diluted in Laemmli sample buffer and loaded into a 0.75-mm-thick polyacrylamide gel with a 4% stacking gel cast on a 12.5% resolving gel. The run was stopped as soon as the front had penetrated 3 mm into the resolving gel so that the whole proteome became concentrated in the stacking/resolving gel interface. Bands were stained with Coomassie Brilliant Blue and excised from the gel. Protein enzymatic cleavage (20 µg) was carried out with trypsin (1:20, *w*/*w*) (Promega, Madison, WI, USA) at 37 °C for 16 h, as previously described [111]. Peptides were purified and concentrated using C18 Zip Tip Solid Phase Extraction (Millipore, Burlington, MA, USA). Peptide mixtures were separated by reverse-phase chromatography using an Eksigent nanoLC ultra 2D pump fitted with a 75-μm ID column (Eksigent 0.075 × 250). Samples were first loaded for desalting and concentration into a 0.5-cm length 100-μm ID precolumn, packed with the same chemicals as the separating column. Mobile phases were 100% water with 0.1% formic acid (FA) (buffer A), and 100% acetonitrile with 0.1% FA (buffer B). The column gradient was developed in a 240-min two-step gradient from 5% B to 25% B in 210 min and 25% B to 40% B in 30 min. The column was equilibrated in 95% B for 9 min and 5% B for 14 min. Throughout the process, the precolumn was in line with the column and the flow was maintained along the gradient at 300 nl/min. Eluting peptides from the column were analyzed with a Sciex 5600 Triple-TOF system (Sciex, Framingham, MA, USA). Data were acquired from a survey scan performed on a mass ranging from 350 to 1250 m/z in a scan time of 250 ms. The top 35 peaks were selected for fragmentation. The minimum accumulation time for MS/MS was set to 100 ms, giving a total cycle time of 3.8 s. Product ions were scanned in a mass range from 230 to 1500 m/z and were excluded from further fragmentation for 15 s.

### 4.12. Peptide Identification and Quantification

MS/MS data were acquired using Analyst 1.7.1 (Sciex, Framingham, MA, USA). Spectra files were processed with Protein Pilot software v.5.0 (Sciex, Framingham, MA, USA) using the Paragon™ algorithm (v.4.0.0.0) for database searching [112], Progroup™ for data grouping, and searched against the concatenated target-decoy UniProt proteome reference human database (Proteome ID: UP000005640, 73,045 proteins, April 2018) plus human papillomavirus type 16 database (Taxon identifier: 333760, nine reviewed proteins, April 2018). Both databases were compiled using Notepad++ software. The data were searched with respect to the fixed modification of carbamidomethyl (C) and the following variable modifications: oxidation (M), deamidation (NQ), glutamine to pyro-glutamate (N-terminus), with a one-missed tryptic cleavage threshold and a 10-ppm mass error tolerance for precursor and 20 ppm for fragment ions. Only spectra with 95% confidence or better were considered. The false-discovery rate (FDR) was estimated by a non-linear fitting method [113] and the results displayed were those reporting a 1% global FDR or better. The peptide was quantified with Progenesis LC−MS software (v.2.0.5556.29015, Nonlinear Dynamics). Runs were aligned to compensate for between-run variations in our nanoLC separation system using the accurate mass measurements from full survey scans in the TOF detector and the observed retention times. All runs were aligned to a reference run that was automatically chosen by the software, and a master list of features considering m/z values and retention times was generated. The quality of these alignments was manually supervised with the help of quality scores provided by the software. The peptide identifications were exported from Protein Pilot software and imported into the Progenesis LC−MS program, where they were matched to the respective features. Output data files were managed with Perseus software [114] to enable statistical analyses and representation. Proteins identified by site (identification based only on a modification), reverse proteins (identified by decoy database) and potential contaminants were filtered out. Principal component analysis was performed on the normalized and filtered datasets. After checking the data were normally distributed, significantly different protein levels were calculated by ANOVA with a permutation-based FDR cut-off (250 randomizations, FDR < 0.05). Proteins quantified with at least two unique peptides, an ANOVA significance of *p* < 0.05, and an absolute < 0.77-fold (downregulation) or > 1.3-fold (upregulation) change in linear scale were considered to be significantly differentially expressed. MS raw data and search results files have been deposited to the ProteomeXchange Consortium (http://proteomecentral.proteomexchange.org accessed on 8 February 2019) via the Proteomics Identifications Database (PRIDE) partner repository [115], with the dataset identifier PXD014993.

### 4.13. Bioinformatic Analysis

The disrupted biological functions triggered by miR-877-3p inhibition were interrogated using several bioinformatic tools: DAVID v6.8 (https://david.ncifcrf.gov/ accessed on 5 November 2018) [116], PANTHER v.14.1 (http://pantherdb.org/ accessed on 5 November 2018) [117] and UniProtKB/Swiss-Prot (https://www.uniprot.org/ accessed on 5 November 2018) databases. Data from each cell line were integrated using the STRING v11.0 tool (https://string-db.org/ accessed on 23 January 2019) [118]. The Ingenuity Pathway Analysis (IPA) program (release date: 2019-02-08, https://www.qiagenbioinformatics.com/products/ingenuity-pathway-analysis accessed on 2 May 2019) (Qiagen, Hilden, Germany) [119] was also used to generate larger networks by adding other functionally related and published proteins that were not detected here. Finally, the Metascape tool (http://metascape.org/) [120] ( accessed on 23 May 2019) was used to integrate functional data from the two cell lines.

### 4.14. Western Blot

To check subcellular fractionation efficiency, cytoplasmic and nuclear proteins were separately extracted from MOCK, NC-transfected and anti-miR-877-3p-transfected C-33A and SiHa cells, as explained above, and subjected to western blot. To confirm that miR-877-3p regulated the expression of some potential targets, p16, ZNF177, GPC1, and CCT3 protein levels were determined by western blot in miR-877-3p-silenced SCCC cell lines. Whole-cell protein extracts were isolated from NC-transfected and anti-miR-877-3p-transfected C-33A and SiHa cells by lysing with 30 µL of radioimmunoprecipitation assay (RIPA) buffer (Sigma-Aldrich, St Louis, MO, USA) and a cocktail of protease inhibitors (Roche, Basel, Switzerland). Proteins were collected from supernatants after centrifuging at 8000× *g* for 10 min at 4 °C. For western blot, 60 μg of protein were resolved by SDS-PAGE in a Criterion™ TGX Stain-Free™ protein gel, and transferred onto a nitrocellulose membrane (both from Bio-Rad, Hercules, CA, USA), which was blocked with 5% non-fat milk. Membranes were incubated with anti-p16 (805–4713, Roche, Basel, Switzerland), anti-ZNF177 (ab50718, Abcam, Cambridge, UK), anti-GPC1, and anti-CCT3 (CSB-PA009703LA01HU and A6547, from Antibodyplus, Inc., Brookline, MA, USA) antibodies at 250 ng/mL, 1:100, 1:500, and 1:500, respectively, overnight and at 4 °C. They were then incubated with secondary anti-rabbit or anti-mouse antibodies (Bio-Rad, Hercules, CA, USA) at 1:2000 for 1 h at room temperature. The signal was detected with the SuperSignal West Pico Chemiluminescent Substrate kit (Thermo Scientific, Rockford, IL, USA) in a ChemiDoc imaging system (Bio-Rad, Hercules, CA, USA) using ImageLab software. The GAPDH (CB1001, Calbiochem, Burlington, MA, USA), histone H3 (ab17684, Abcam, Cambridge, UK), and α-tubulin (T-6074 from Sigma-Aldrich, St Louis, MO, USA) antibodies were employed as loading controls for cytoplasmic, nuclear and whole-cell protein fractions, respectively. Finally, the intensity of bands was quantified by densitometric analysis using the ImageJ program. All whole blots showing all the bands, molecular markers and complete quantifications are shown in the Figure S12.

### 4.15. Immunohistochemistry

Four-µm sections of FFPE cervical lesions were placed on slides and deparaffinized, hydrated and treated to block endogenous peroxidase activity. After incubating with anti-ZNF177 antibody (HPA003141, Sigma-Aldrich, St Louis, MO, USA) at 1:60 for 1 h (antigen retrieval at 90 °C for 30 min, pH 6.0), the signal was developed using a Bond Polymer Refine Detection kit (Leica, Wetzlar, Germany) and visualized with diaminobenzidine. Sections of an FFPE testis were also included as a positive control of ZNF177 staining, as recommended by the antibody manufacturers. The pattern and intensity of expression were evaluated blind by two independent observers (T.Z. and E.M.-S.), and cases were ascribed to one of four categories: 0, no expression; 1, weak expression; 2, moderate expression; 3, strong expression (Figure S13). Finally, an immunohistochemical score was defined for each sample as the mean of the observers’ evaluation. Images were acquired with a Leica DM4000B microscope (Leica, Wetzlar, Germany) and the NIS Elements program (Nikon Instruments, Amsterdam, The Netherlands) at 200× and 400× magnifications.

### 4.16. Response to Paclitaxel

To determine whether miR-877-3p was involved in response to paclitaxel, CC cell sensitivity to paclitaxel was first evaluated. To do this, C-33A, SiHa and HeLa cells were seeded in 96-well plates at a density of 1 × 10^4^ cells/well, allowed to attach overnight, and treated with a wide range of paclitaxel (Selleck Chemicals, Houston, TX, USA) doses for 72 h, using DMSO (Sigma-Aldrich, St Louis, MO, USA) as vehicle control. Cell viability was measured as the intracellular ATP content using the CellTiter-Glo^®^ luminescent cell viability assay (Promega, Madison, WI, USA), and IC_50_ values were calculated as previously described [121]. Next, miR-877-3p silencing and paclitaxel treatment were carried out simultaneously, and cell morphology, viability, and migration were monitored under a Leica DMi1 microscope for 24, 48, and 72 h, as described above. Images were taken at each time with the Leica Application Suite v4.12 program (Leica, Wetzlar, Germany) at 50× and 200× magnifications. The nature of the combination was mathematically determined using the Bliss independence principle [122,123], which enabled the calculation of the predicted inhibitory effect of the double targeting based on the inhibitory effect observed after individual administration, under the assumption that drugs acted independent of each other, as:

PE_combination_ = OE_anti-miR-877-3p_ + OE_paclitaxel_ − OE_anti-miR-877-3p_ × OE_paclitaxel_

where PE_combination_ is the predicted effect of the combination; OE_anti-miR-877-3p_ is the observed effect of the anti-miR-877-3p; and OE_paclitaxel_ is the observed effect of paclitaxel. If the observed inhibitory effect of the drug combination (OE_combination_) is equal the PE_combination_, drugs act independently; if the OE_combination_ is greater than the PE_combination_, the combination is considered as synergistic; and if the OE_combination_ is lower than the PE_combination_, the combination is antagonistic.

### 4.17. Statistical Analysis

Patients’ demographic, clinical, pathological, and molecular data were summarized as frequencies (and percentages) and means (and ranges) ± standard error of the mean, as appropriate. Unless otherwise specified, all in vitro experiments were carried out in three biological replicates from three CC cell lines. All statistical analyses, except those involving bioinformatics (detailed above), were carried out using IBM SPSS Statistics v25. Group differences of each variable were compared by Student’s independent samples t-test or the Mann–Whitney test, as appropriate. Associations among variables were estimated by χ^2^ or Fisher’s exact tests. Kaplan–Meier plots and log-rank tests were used to examine the association of molecular biomarker expression and subcellular location with progression-free survival and overall survival. A multivariate Cox regression model was fitted to test the independent contribution of each variable to patient’s outcome after adjustment. Hazard ratios and 95% confidence intervals were used to estimate the effect of each variable on the outcome. Sensitivity and specificity of ZNF177 subcellular location in predicting prognosis and diagnosis was calculated by performing receiver operating curve analysis.

## 5. Conclusions

In conclusion, our findings as a whole suggest tools that could improve the clinical management of CC, based on an understanding of the miR-877-3p molecular mechanism. First, miR-877-3p inhibition could be a therapeutic strategy that impairs CC cell migration and invasion through cytoskeletal protein remodeling and cooperation with paclitaxel. Second, the expression of one of its targets, ZNF177, could be a potential diagnostic biomarker distinguishing HSIL from SCCC. Finally, cytoplasmic ZNF177 levels potentially predict poor prognosis in SCCC patients.

## Figures and Tables

**Table 1:** Distribution of miR-877-3p expression in our series of cervical samples. Number and percentage of patients with miR-877-3p levels below and above the median expression of the whole series. (BL: benign lesion of the cervical epithelium; HSIL: high-grade squamous intraepithelial lesion; SCCC: squamous cell carcinoma of the cervix; NA: not analyzable due to small quantity or poor quality of RNA).

Cervical Tissue	miR-877-3p Expression		
	≤Median	>Median	NA
BL	18 (78%)	2 (9%)	3 (13%)
HSIL	20 (48%)	18 (43%)	4 (9%)
SCCC	14 (27%)	32 (62%)	6 (11%)

**Table 2:** Distribution of ZNF177 protein expression and subcellular location in our series of cervical tissues. Number and percentage of patients with negative (0), low (1), moderate (2), and high (3) levels of expression of nuclear and cytoplasmic ZNF177 protein levels, showing a very significant association with the cervical lesion type (*p* = 0.001 and 0.004, respectively). (BL: benign lesion of the cervical epithelium; HSIL: high-grade squamous intraepithelial lesion; SCCC: squamous cell carcinoma of the cervix; NA: not analyzable due to limited representativeness of the tissue slice).

Cervical Tissue	Nuclear ZNF177					Cytoplasmic ZNF177				
	0	1	2	3	NA	0	1	2	3	NA
BL	7 (30%)	13 (57%)	1 (4%)	2 (9%)	0 (0%)	19 (83%)	4 (17%)	0 (0%)	0 (0%)	0 (0%)
HSIL	19 (45%)	12 (29%)	2 (5%)	0 (0%)	9 (21%)	10 (24%)	20 (48%)	3 (7%)	0 (0%)	9 (21%)
SCCC	34 (65%)	8 (15%)	0 (0%)	0 (0%)	10 (19%)	24 (46%)	15 (29%)	3 (6%)	0 (0%)	10 (19%)

**Table 3:** Nature of the anti-miR-877-3p + paclitaxel combination in CC cell lines. Inhibitory effect on CC cell viability, calculated as 1 minus the proportion of viable cells in silenced or treated cells vs. NC- and DMSO-treated cells, represented in Figure 6B and Figure S10B. Inhibitory effect on CC cell migration ability, calculated as 1 minus the proportion of migration index in silenced or treated cells vs. NC- and DMSO-treated cells, represented in Figure 6C and Figure S10C. The predicted effect of the combination (PE_combination_) was calculated as: PE_combination_ = OE_anti-miR-877-3p_ + OE_paclitaxel_ − OE_anti-miR-877-3p_ × OE_paclitaxel_; where OE_anti-miR-877-3p_ is the observed effect of the anti-miR-877-3p; and OE_paclitaxel_ is the observed effect of paclitaxel.

Effect	Drug	C-33A		SiHa		HeLa	
		NC	anti-miR-877-3p	NC	anti-miR-877-3p	NC	anti-miR-877-3p
Cell viability	DMSO	0.00	0.18	0.00	0.24	0.00	0.17
	Paclitaxel	0.67	0.67	0.16	0.40	0.14	0.36
	PE_combination_	0.73		0.37		0.29	
Cell migration	DMSO	0.00	0.54	0.00	0.77	0.00	0.23
	Paclitaxel	0.92	1.52	0.26	1.10	−0.17	0.97
	PE_combination_	0.96		0.83		0.09	

## Data Availability

The data presented in this study are available in this article (and Supplementary Materials).

## Supplementary Materials

The following are available online at https://www.mdpi.com/article/10.3390/cancers13071739/s1. Table S1: Summary of information regarding identified peptides and proteins. Table S2: miR-877-3p-regulated protein quantification by LC–MS/MS in C-33A cells. Table S3: miR-877-3p-regulated protein quantification by LC–MS/MS in SiHa cells. Table S4: The five molecular and cellular functions most significantly deregulated by miR-877-3p in C-33A cells. Table S5: The five molecular and cellular functions most significantly deregulated by miR-877-3p in SiHa cells. Table S6: Pathological and clinical characteristics of our series of 52 SCCC patients. Figure S1: Effects of miR-877-3p silencing on HeLa cells. Figure S2: Effects of miR-877-3p silencing on early events in CC cell survival. Figure S3: ZNF177 expression in CC tissues. Figure S4: Correlation between miR-877-3p and ZNF177 expression in CC tissues. **Figure S5**: Association between cytoplasmic ZNF177 levels and SCCC patients’ overall survival. Figure S6: Accuracy of ZNF177 subcellular location as potential biomarker in cervical tissues. Figure S7: Proteins significantly altered by miR-877-3p in SCCC cell lines. Figure S8: Networks most highly deregulated by miR-877-3p in SCCC cells. Figure S9: Paclitaxel treatment in CC cell lines. Figure S10: Simultaneous miR-877-3p inhibition and paclitaxel treatment in HeLa cells. Figure S11: Subcellular fractionation efficiency. Figure S12: Whole images and densitometric quantification of each western blot. Figure S13: Representative images of each immunohistochemical score for ZNF177 expression in several tissues.
