# Supplementary material for: Understanding the Molecular Mechanism of miR-877-3p Could Provide Potential Biomarkers and Therapeutic Targets in Squamous Cell Carcinoma of the Cervix
Source: Cancers (Basel). 2021 Apr 6;13(7):1739. doi: 10.3390/cancers13071739 (PMC8038805; doi:10.3390/cancers13071739)
Supplement: Supplementary file 1 [file cancers-13-01739-s001.zip › Supplementary Information_proofreading.docx]

**Understanding the molecular mechanism of miR-877-3p could provide potential biomarkers and therapeutic targets in squamous cell carcinoma of the cervix**

Saioa Mendaza, Joaquín Fernández-Irigoyen, Enrique Santamaría, Imanol Arozarena, David Guerrero-Setas, Tamara Zudaire, Rosa Guarch, August Vidal, José-Santos Salas, Xavier Matias-Guiu, Karina Ausín, Carmen Gil, Rubén Hernández-Alcoceba, Esperanza Martín-Sánchez^*^

**Supplementary Information**

The supplementary information consists of 6 tables and 13 figures.

**Supplementary Tables**

**Supplementary Table S1.** Summary of all peptide sequences assigned in each differential experiment, including mass, peptide sequence, peptide score, and modifications; and summary of all proteins identified in each experiment, including protein groups, accessions and peptides assigned.

**Supplementary Table S2. miR-877-3p-regulated protein quantification by LC-MS/MS in C-33A cells.** The most abundant proteins were detected and quantified in the cytoplasm and nucleus of MOCK, NC-transfected and anti-miR-877-transfected C-33A cells by LC-MS/MS. Of these, proteins fulfilling our criteria for identification (see Material & Methods section) are summarized in the “Quantified proteins” sheets. Proteins significantly regulated by miR-877-3p were identified by statistical comparison between anti-miR-877-3p and NC conditions (“Differential proteins” sheets).

**Supplementary Table S3. miR-877-3p-regulated protein quantification by LC-MS/MS in SiHa cells.** The most abundant proteins were detected and quantified in the cytoplasm and nucleus of MOCK, NC-transfected and anti-miR-877-transfected SiHa cells by LC-MS/MS. Of these, the proteins fulfilling our criteria for identification (see Material & Methods section) are summarized in the “Quantified proteins” sheets. Proteins significantly regulated by miR-877-3p were identified by statistical comparison between anti-miR-877-3p and NC conditions (“Differential proteins” sheets).

**Supplementary Table S4. The five molecular and cellular functions most significantly deregulated by miR-877-3p in C-33A cells.** The IPA software suggested functions that could be altered by miR-877-3p in C-33A cells, by integrating proteins identified in our MS/MS experiment with published data.

| **Molecular and cellular function** | **Range of values of *p*** | **Number of molecules** |
| --- | --- | --- |
| Protein synthesis | 7.05E-10 – 1.65E-19 | 55 |
| Cellular assembly and organization | 1.01E-07 – 1.23E-10 | 15 |
| Post-translational modification | 1.82E-10 – 1.83E-10 | 12 |
| Protein folding | 1.83E-10 – 1.83E-10 | 12 |
| Protein degradation | 7.05E-10 – 7.05E-10 | 15 |

**Supplementary Table S5. The five molecular and cellular functions most significantly deregulated by miR-877-3p in SiHa cells.** The IPA software suggested functions that could be altered by miR-877-3p in SiHa cells, by integrating proteins identified in our MS/MS experiment with published data.

| **Molecular and cellular function** | **Range of values of *p*** | **Number of molecules** |
| --- | --- | --- |
| Cellular compromise | 4.55E-05 – 6.37E-10 | 25 |
| Cellular development | 2.22E-06 – 1.29E-09 | 38 |
| Cellular growth and proliferation | 2.22E-06 – 1.29E-09 | 38 |
| Cell death and survival | 2.82E-06 – 1.59E-09 | 61 |
| Protein synthesis | 5.93E-07 – 4.04E-09 | 26 |

**Supplementary Table S6. Pathological and clinical characteristics of our series of 52 SCCC patients.** (NA: not available).

| **Feature** | **Frequency (%)** |
| --- | --- |
| **Age** (years) | Mean: 54  Range: 30-82 |
| **Grade** |  |
| 1 | 16 (31) |
| 2 | 14 (27) |
| 3 | 14 (27) |
| NA | 8 (15) |
| **Lymph node involvement** |  |
| No | 37 (71) |
| Yes | 12 (23) |
| NA | 3 (6) |
| **Vascular invasion** |  |
| No | 24 (46) |
| Yes | 25 (48) |
| NA | 3 (6) |
| **Stage** |  |
| I | 29 (56) |
| II | 6 (11) |
| III | 9 (17) |
| IV | 5 (10) |
| NA | 3 (6) |
| **HR-HPV status** |  |
| Negative | 0 (0) |
| Positive | 52 (100) |
| **Progression-free survival** (months) | Mean: 121  Range: 3-263 |
| Not relapsed | 37 (71) |
| Relapsed | 15 (29) |
| **Overall survival** (months) | Mean: 129  Range: 5-263 |
| Exitus | 18 (35) |
| **Therapy** |  |
| Only chemotherapy | 0 (0) |
| Only radiotherapy | 16 (31) |
| Chemotherapy+ radiotherapy | 24 (46) |
| NA | 12 (23) |

**Supplementary Figures**


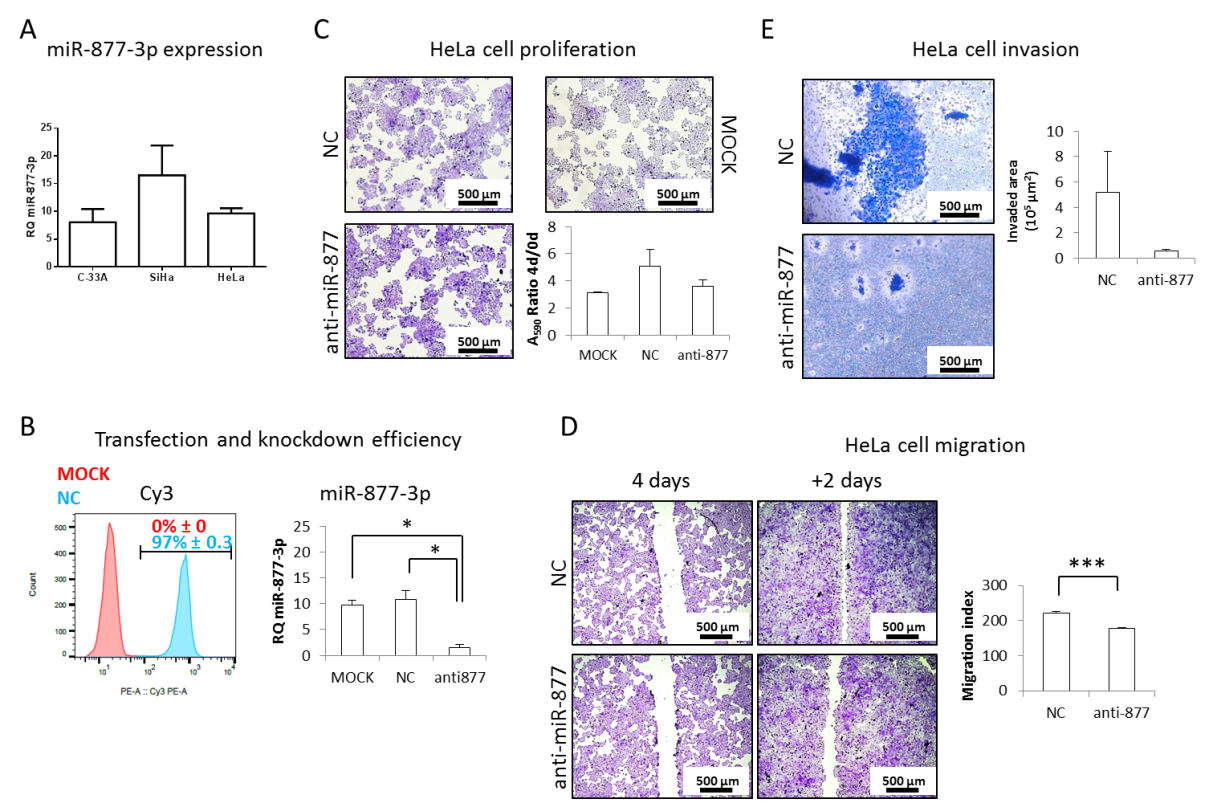


**Supplementary Figure S1. Effects of miR-877-3p silencing on HeLa cells. (A)** miR-877-3p was measured by qRT-PCR in all three CC cell lines. **(B)** HeLa cells were transfected with a Cy3-labelled negative control anti-miR (NC) and an anti-miR-877-3p. Transfection and knockdown efficiencies were checked by flow cytometry (left panel) and qRT-PCR (right panel), respectively. HeLa **(C)** cell proliferation, **(D)** migration and **(E)** invasion were analyzed after 2 and 3 days, respectively, following a 4-day transfection. Cells were stained with crystal violet and absorbance was measured at 590 nm. The migration index was calculated as the scratch width at 4 days minus that at 4+2 days. All images were acquired at 50X magnification. (*, *p* < 0.05; ***, *p* < 0.001; RQ, relative quantification).


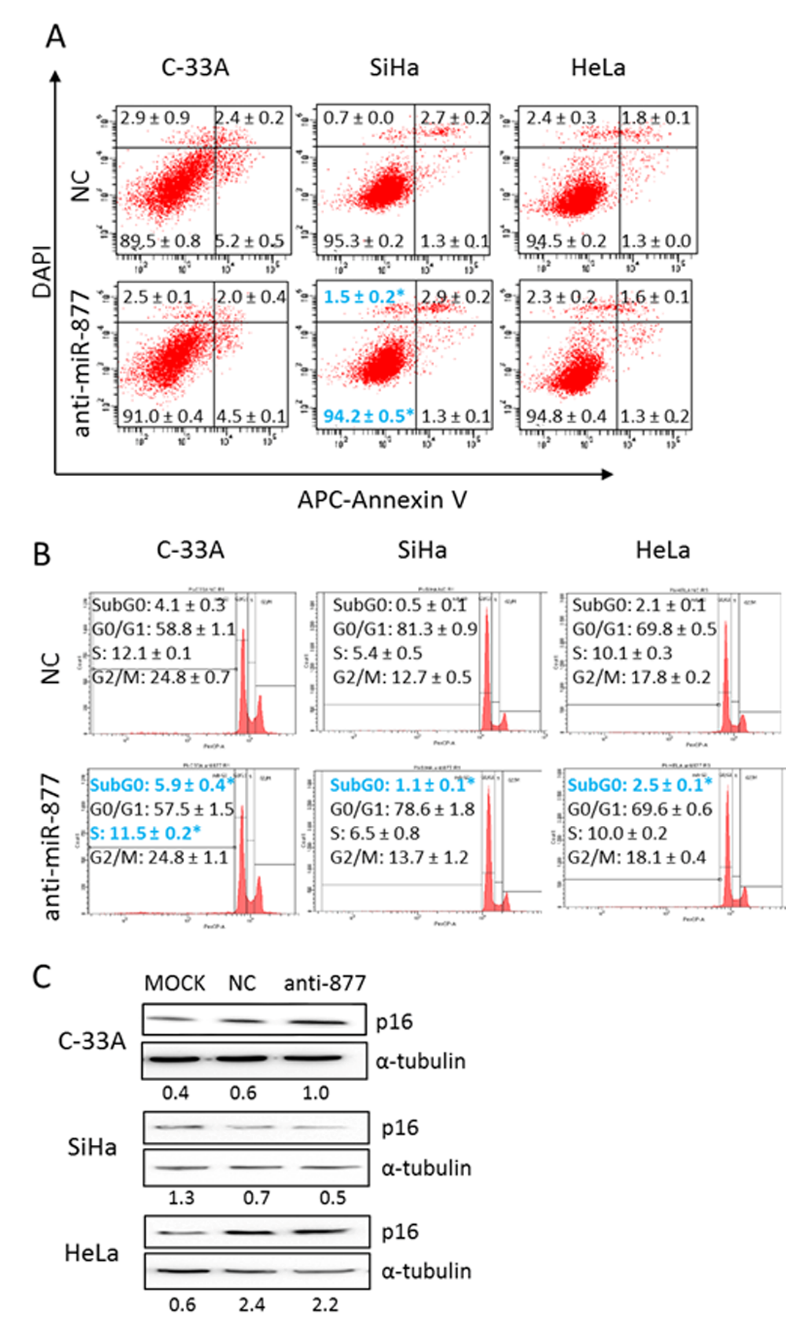


**Supplementary Figure S2. Effects of miR-877-3p silencing on early events in CC cell survival.** C-33A, SiHa and HeLa cell lines were transfected with the negative control anti-miR (NC) and the anti-miR-877-3p, and survival parameters were estimated: **(A)** apoptosis induction was measured by flow cytometry using dual APC-Annexin V and DAPI staining; **(B)** distribution of cell cycle phases was determined by flow cytometry with propidium iodide staining; and **(C)** p16 expression was assessed by western blot. Blue numbers in panels A and B highlight significant differences in anti-miR-877 with respect to NC (*, *p*<0.05).


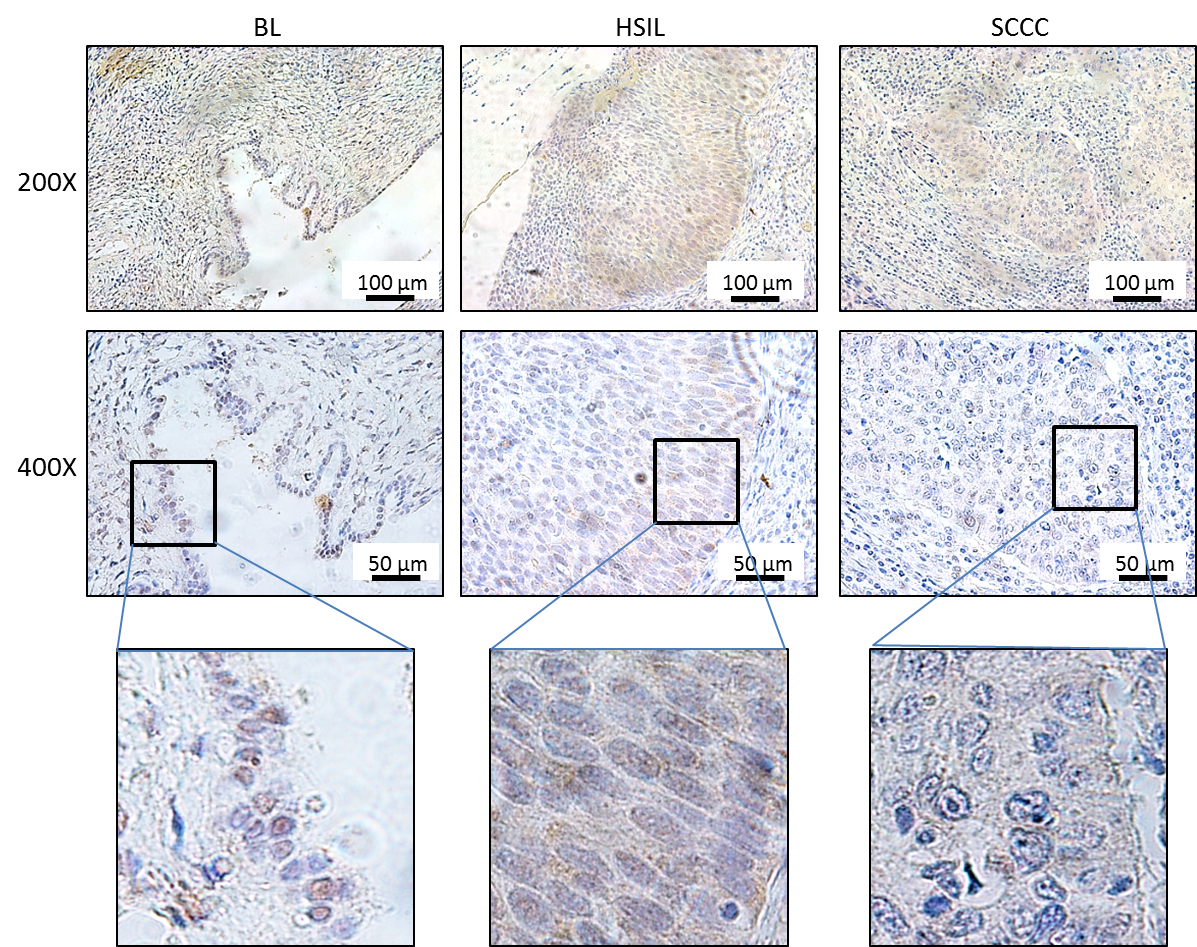


**Supplementary Figure S3. *ZNF177* expression in CC tissues.** Representative images of the ZNF177 immunohistochemical pattern in benign lesions (BLs), high-grade squamous intraepithelial lesions (HSILs) and squamous cell carcinoma of the cervix (SCCCs), acquired at 200X and 400X magnifications. They were scored as 1, 0.25 and 0 for nuclear ZNF177 expression, and as 0, 0.75 and 0.5 for ZNF177 cytoplasmic expression, respectively. Details highlight the mainly nuclear expression of ZNF177 in BLs, and a predominantly cytoplasmic pattern in HSILs and SCCCs.


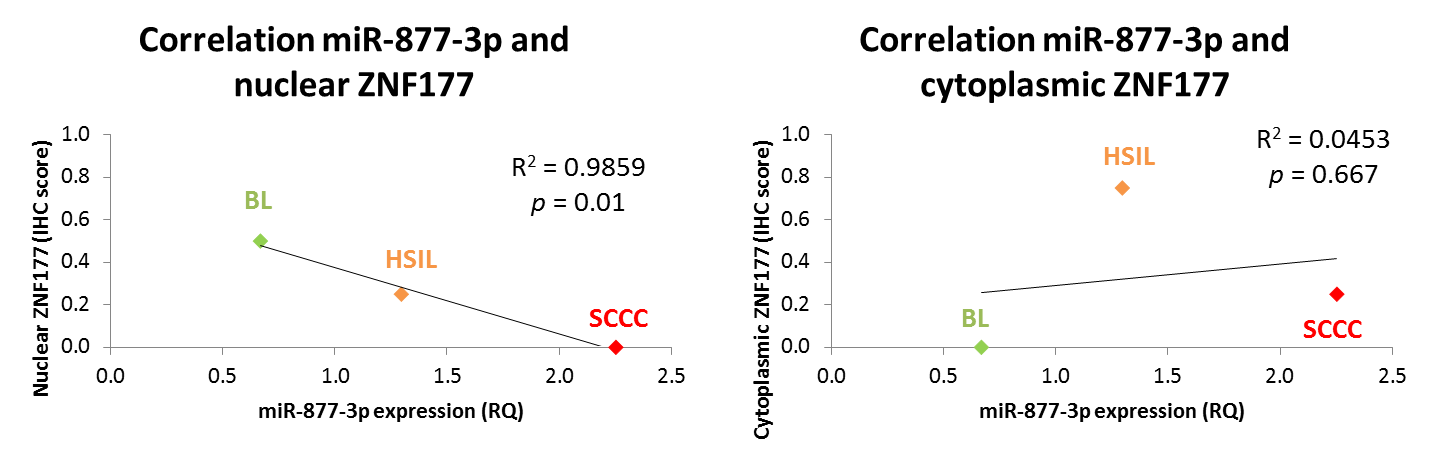


**Supplementary Figure S4. Correlation between miR-877-3p and ZNF177 expression in CC tissues.** miR-877-3p and ZNF177 levels were measured by qRT-PCR and immunohistochemistry, respectively, in benign lesions (BLs) of the cervical epithelium, high-grade squamous intraepithelial lesions (HSILs) and squamous cell carcinoma of the cervix (SCCC).


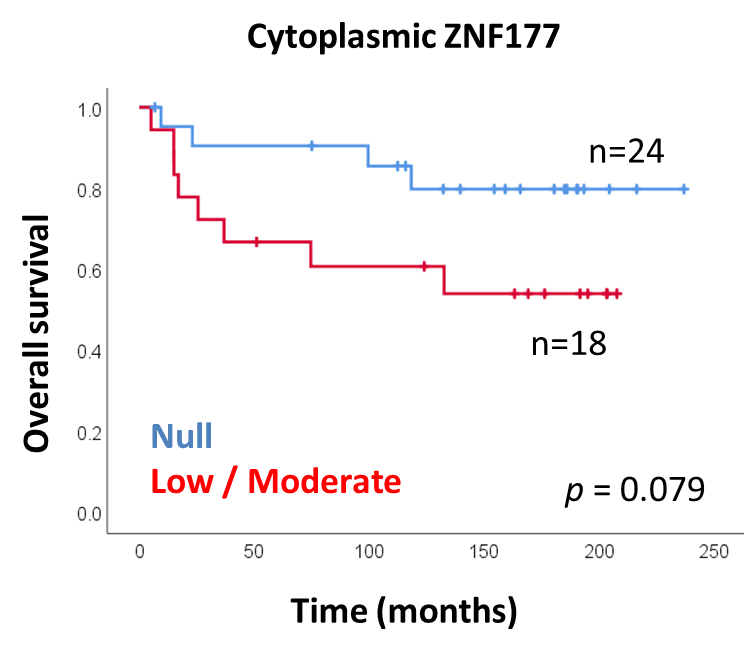


**Supplementary Figure S5. Association between cytoplasmic ZNF177 levels and SCCC patients’ overall survival.**  Cytoplasmic expression of ZNF177 was evaluated by immunohistochemistry in SCCC patients.


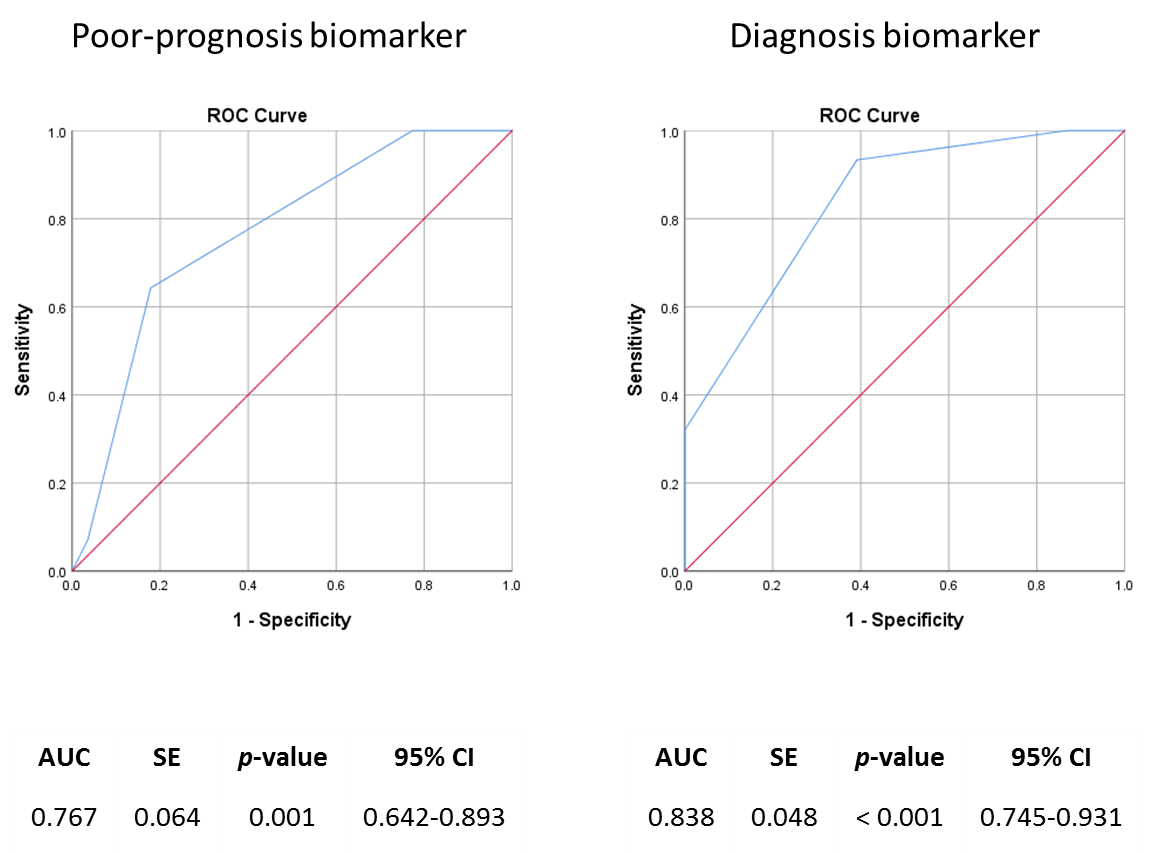


**Supplementary Figure S6. Accuracy of ZNF177 subcellular location as biomarker in cervical tissues.** The receiver operating curves were used to estimate sensitivity and specificity of cytoplasmic ZNF177 levels in predicting prognosis and diagnosis in cervical tissue (AUC, area under the curve; SE, standard error; CI, confidence interval).

**
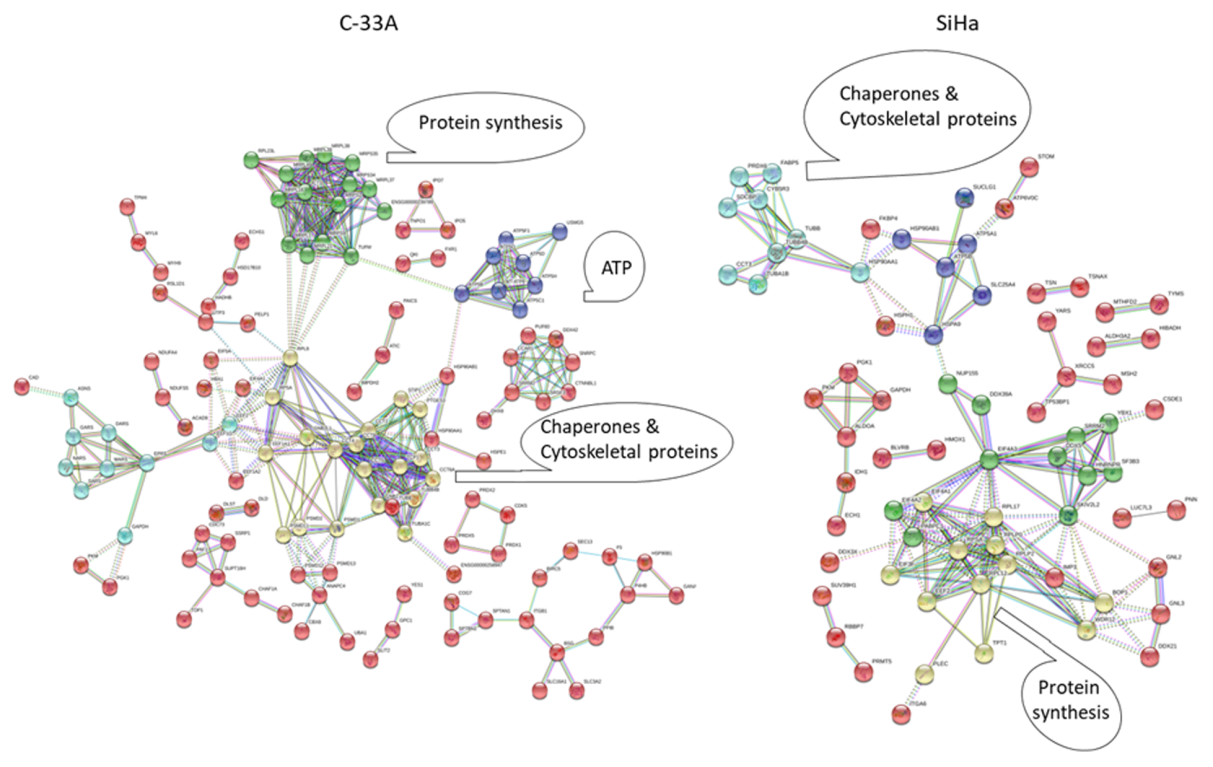
**

**Supplementary Figure S7. Proteins significantly altered by miR-877-3p in SCCC cell lines.** Upon miR-877-3p silencing, protein extracts from NC-transfected and anti-miR-877-transfected cells were subjected to LC-MS/MS. 180 and 114 proteins were significantly deregulated in C-33A and SiHa cells, respectively, upon miR-877 knockdown. Protein networks were identified by the STRING tool, and functional clusters were identified using PANTHER and DAVID tools.


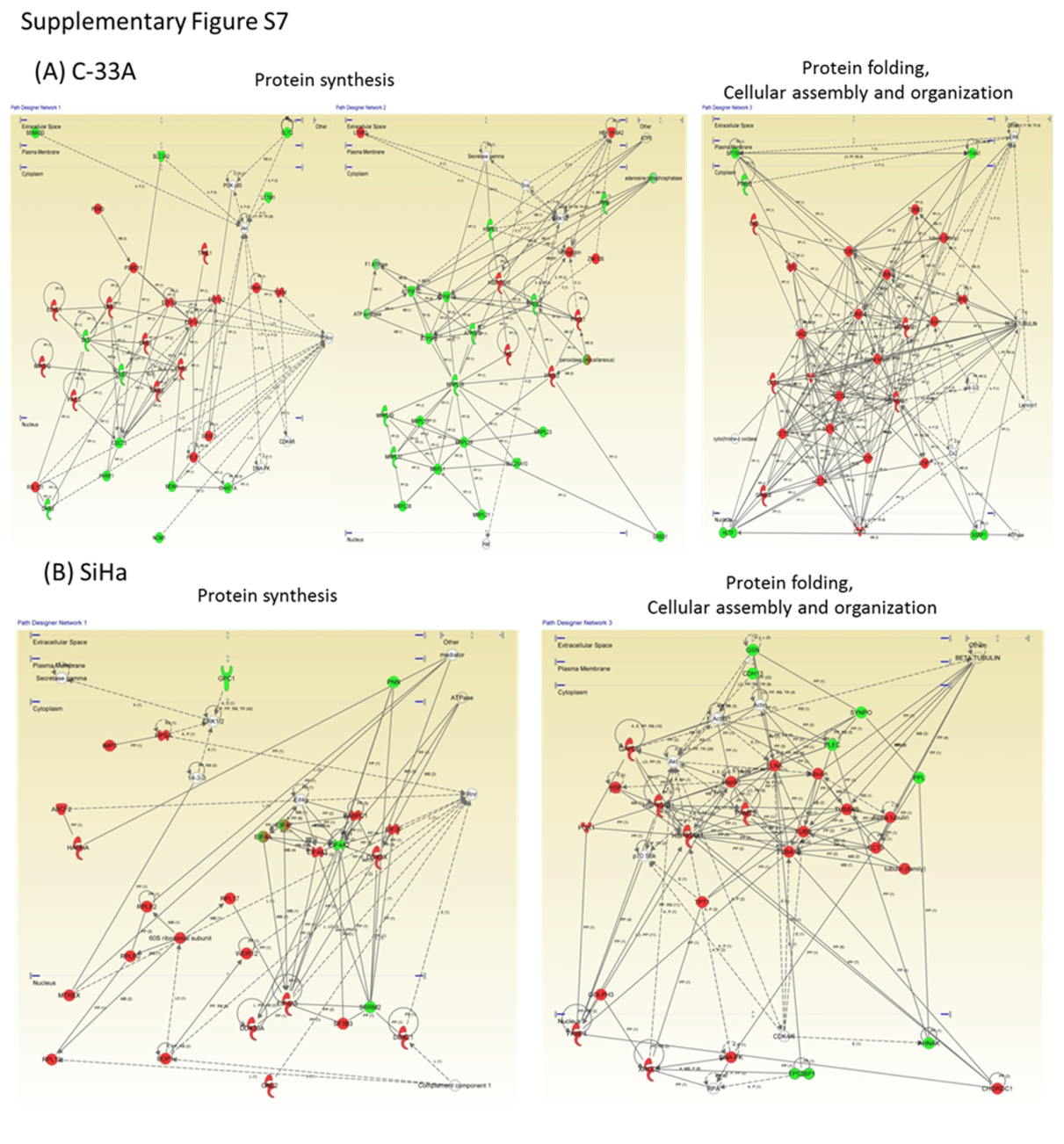


**Supplementary Figure S8. Networks most highly deregulated by miR-877-3p in SCCC cells.** Protein networks involved in cellular and mitochondrial protein synthesis (left panels) and protein folding and cellular assembly (right panels) were indicated by IPA as the networks most significantly deregulated by miR-877-3p in **(A)** C-33A and **(B)** SiHa cells. Red and green circles represent up-regulated and down-regulated proteins in our MS/MS experiment, respectively; white circles indicate proteins not identified in our experiments, but known to be functionally related to them.





**Supplementary Figure S9. Paclitaxel treatment in CC cell lines.** C-33A, SiHa and HeLa cells were treated with several doses of paclitaxel for 72h, after which, cell viability was measured. Data were fitted to a sigmoidal curve, and IC_50_ values were calculated.


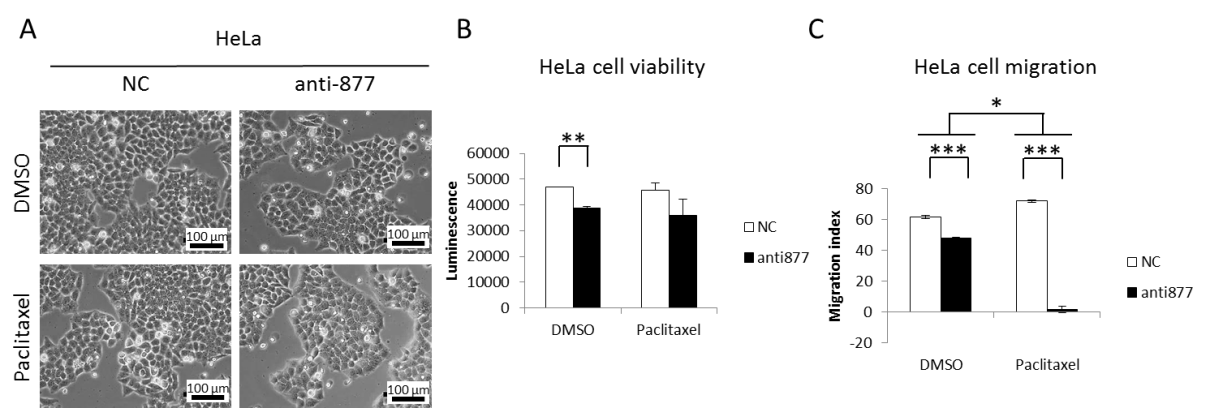


**Supplementary Figure S10. Simultaneous miR-877-3p inhibition and paclitaxel treatment in HeLa cells.** Anti-miR-877-3p transfection and sub-IC_50_ paclitaxel treatment were performed in the HeLa cell line, and effects on cell morphology, viability and migration were monitored for 48 and 72 h. **(A)** Images were captured at 200X magnification after 72 h of double-targeting. **(B)** Cell viability was measured at 72 h as the intracellular ATP content. **(C)** Cell migration was examined at 48 h. The migration index was calculated as the scratch width at time 0 minus that at 48 h (*, *p* < 0.05; **, *p* < 0.01; ***, *p* < 0.001).


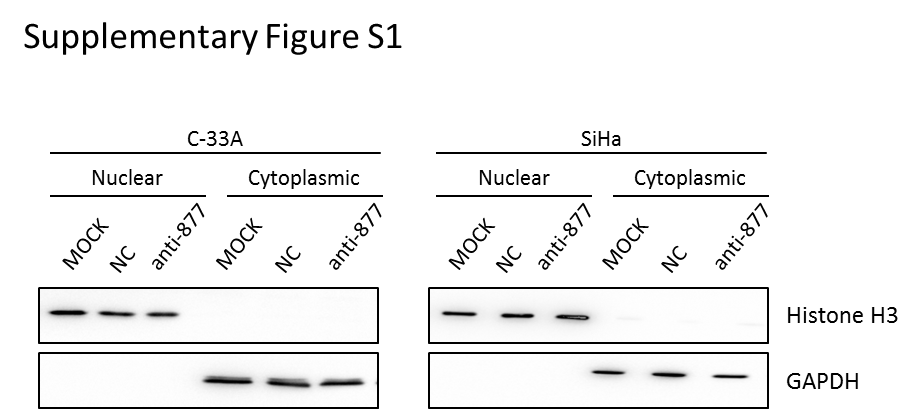


**Supplementary Figure S11. Subcellular fractionation efficiency.** Nuclear and cytoplasmic protein fractions were separately extracted from C-33A and SiHa cells upon miR-877-3p knockdown. Enrichment efficiency was checked by measuring histone H3 and GAPDH levels, respectively, by western blot.

**
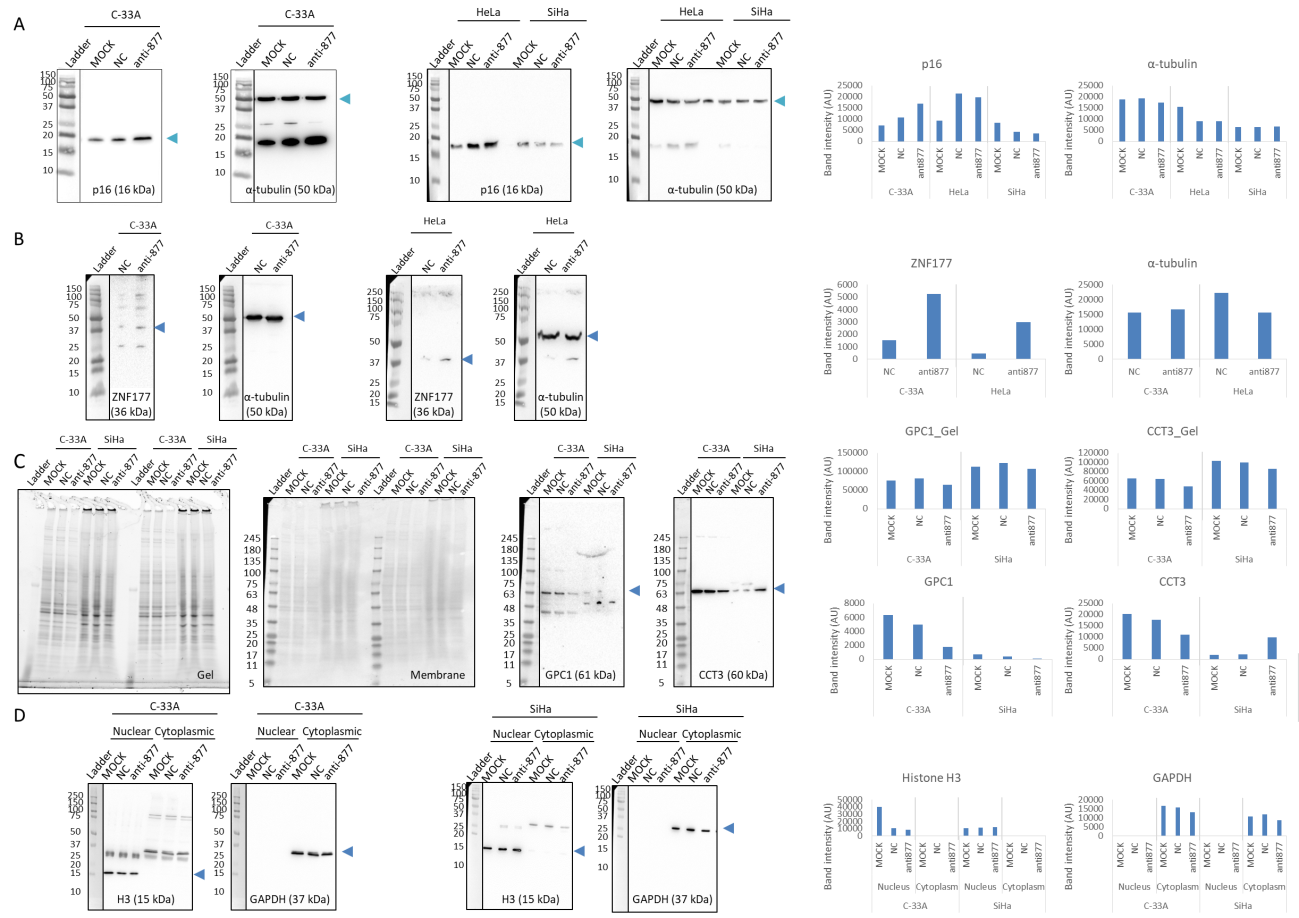
**

**Supplementary Figure S12. Whole images and densitometric quantification of each western blot. (A)** p16 protein levels in C-33A, HeLa and SiHa non-transfected cells (MOCK) and transfected with the anti-miR scramble negative control (NC) or the anti-miR-877-3p. The cropped image is shown in Supplementary Figure S2C. **(B)** ZNF177 expression in C-33A and HeLa cells after transfection with the NC or the anti-miR-877-3p. The cropped image is shown in Figure 4D. **(C)** Entire stain-free gel and membrane before (left panels) and after (right panels) incubation with anti-GPC1 and anti-CCT3 antibodies. The cropped image is shown in Figure 5D. **(D)** Histone H3 and GADPH levels were examined in nuclear and cytoplasmic fractions in C-33A and SiHa cells to show the efficiency of subcellular fractionation procedures. The cropped image is shown in Supplementary Figure S11. In all panels, blue arrowheads indicate the bands of interest, and the intensity of each band is shown in the histograms.

**
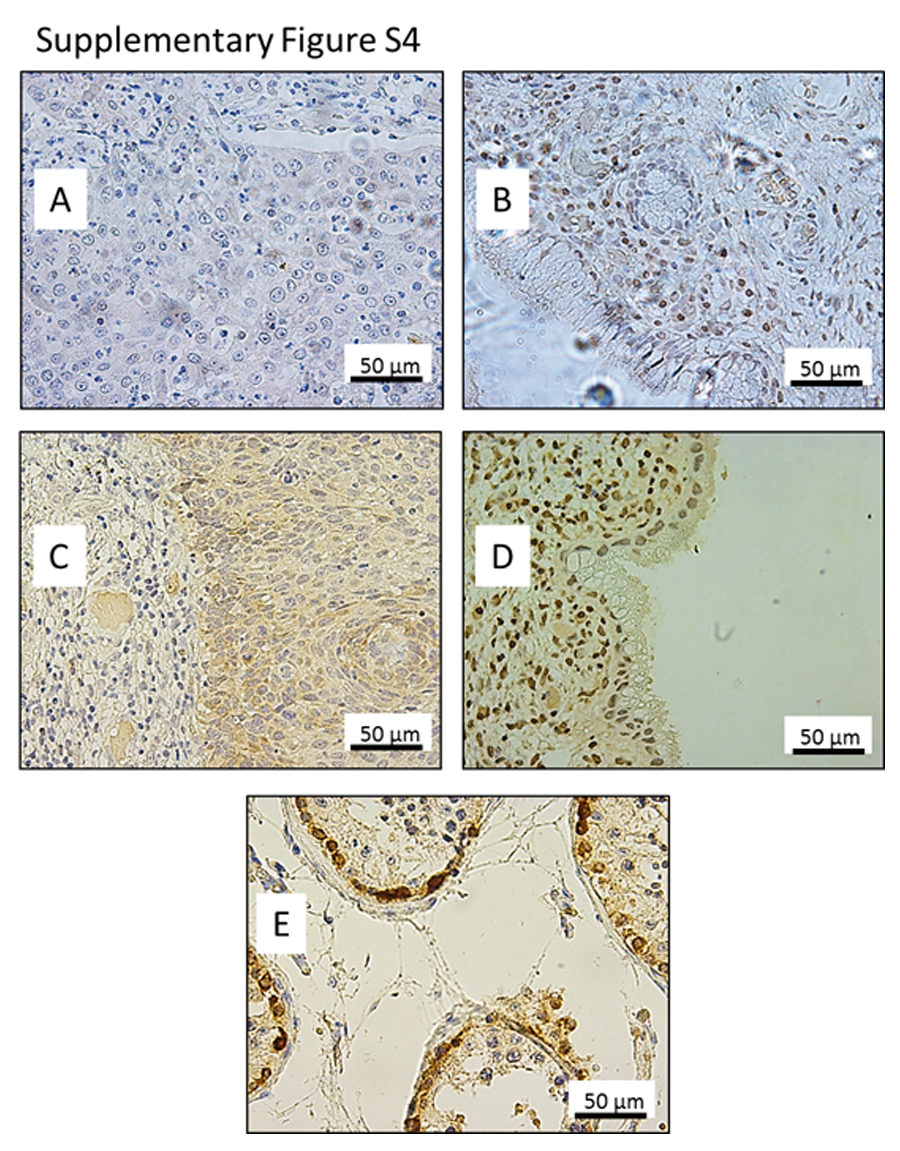
**

**Supplementary Figure S13. Representative images of each immunohistochemical (IHC) score for ZNF177 expression in several tissues. (A)** SCCC tissue with IHC score 0 (no nuclear and no cytoplasmic expression). **(B)** Benign lesion of the cervical epithelium with IHC score 1 (1 and 0 for nuclear and cytoplasmic expression). **(C)** High-grade intraepithelial lesion of the cervix with IHC score 2 (0 and 2 for nuclear and cytoplasmic expression). **(D)** Benign lesion of the cervix with IHC score 2 (2 and 0 for nuclear and cytoplasmic expression). **(E)** Testis with IHC score of 3 used as positive control of staining (0-1 and 3 for nuclear and cytoplasmic expression). Images were acquired at 400X magnification.
