# Supplementary figures and images for: Understanding the Molecular Mechanism of miR-877-3p Could Provide Potential Biomarkers and Therapeutic Targets in Squamous Cell Carcinoma of the Cervix
Source: Cancers (Basel). 2021 Apr 6;13(7):1739. doi: 10.3390/cancers13071739 (PMC8038805; doi:10.3390/cancers13071739)

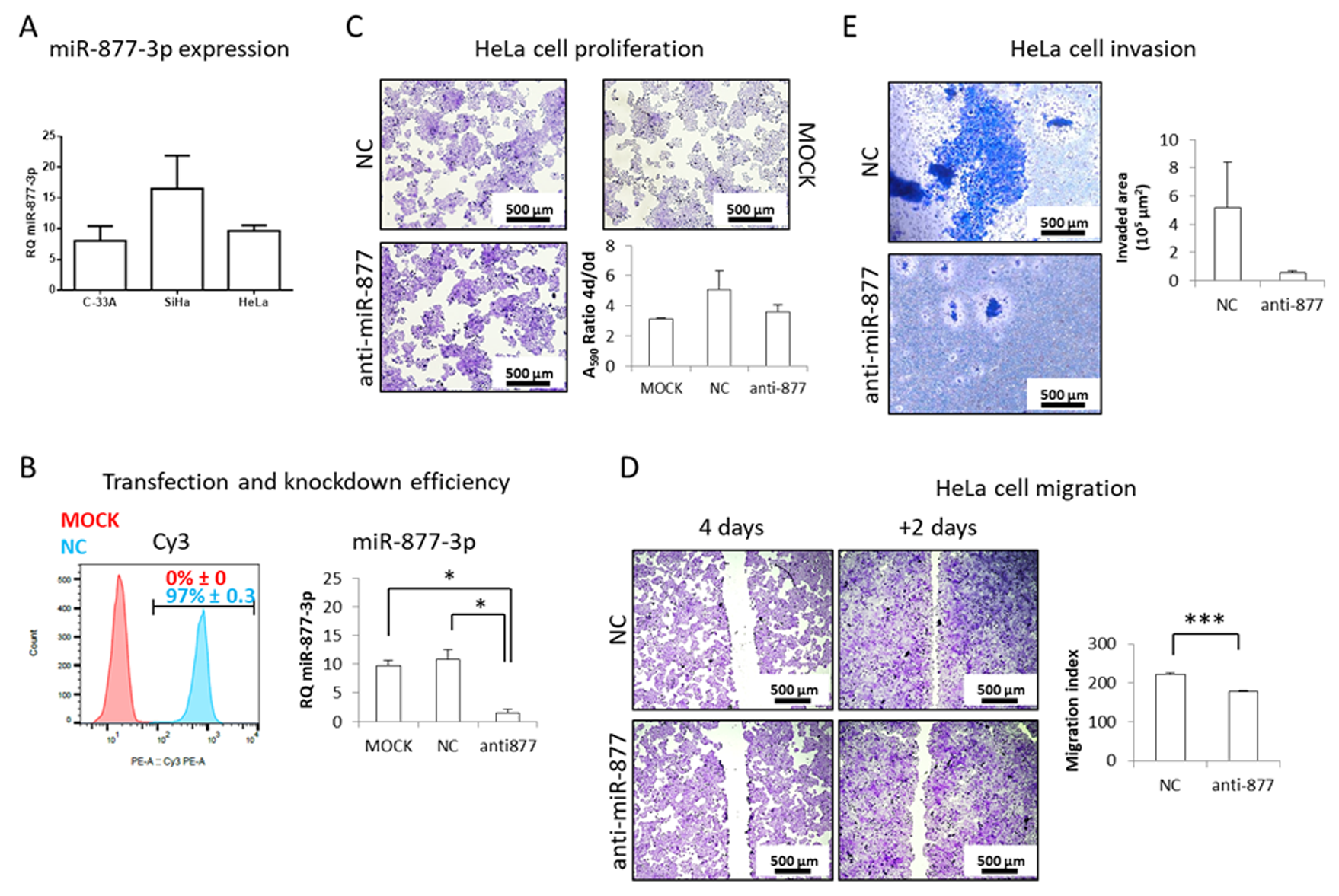

Supplement: Supplementary file 1 [file cancers-13-01739-s001.zip › Supplementary Figure S1.tif]

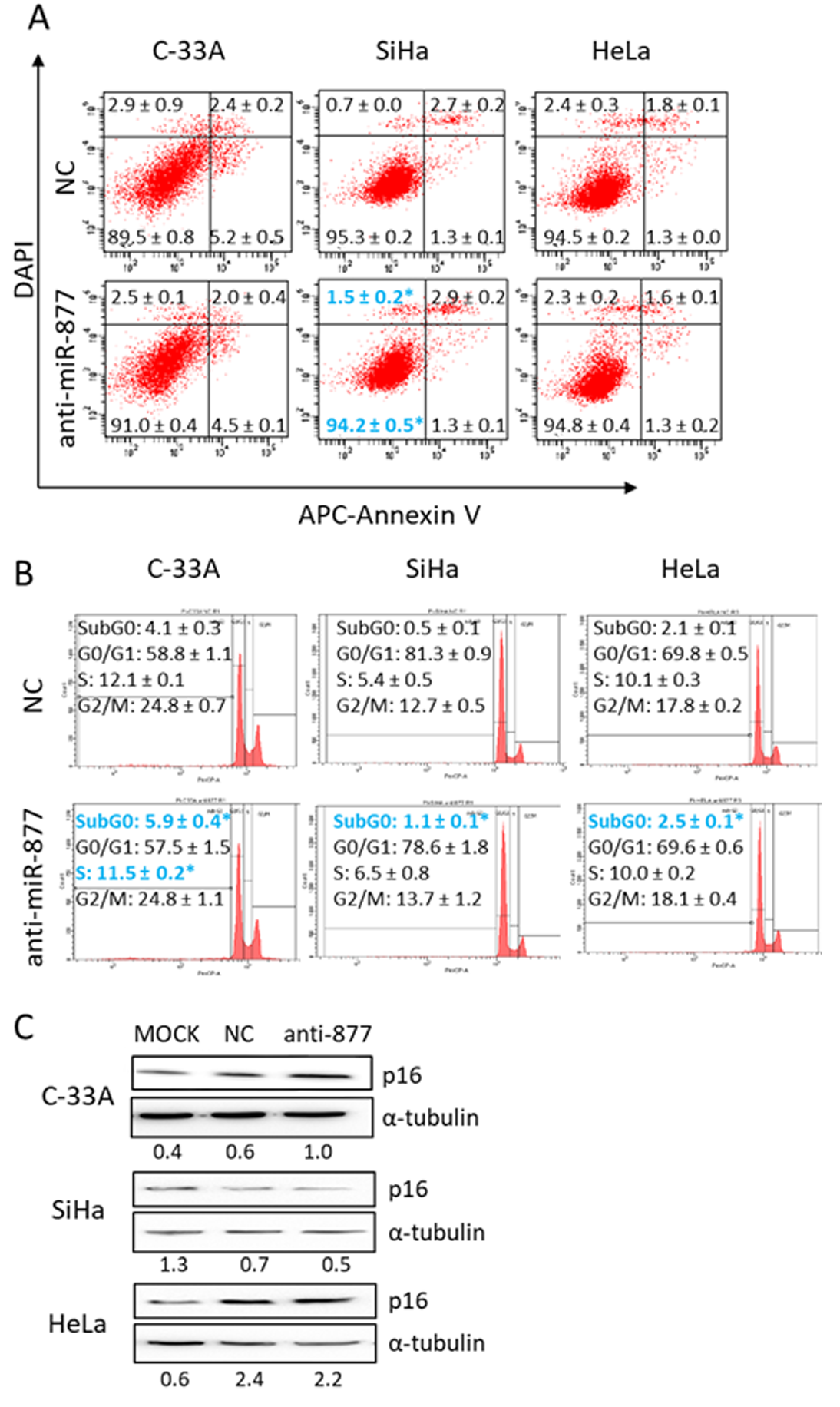

Supplement: Supplementary file 1 [file cancers-13-01739-s001.zip › Supplementary Figure S2.tif]

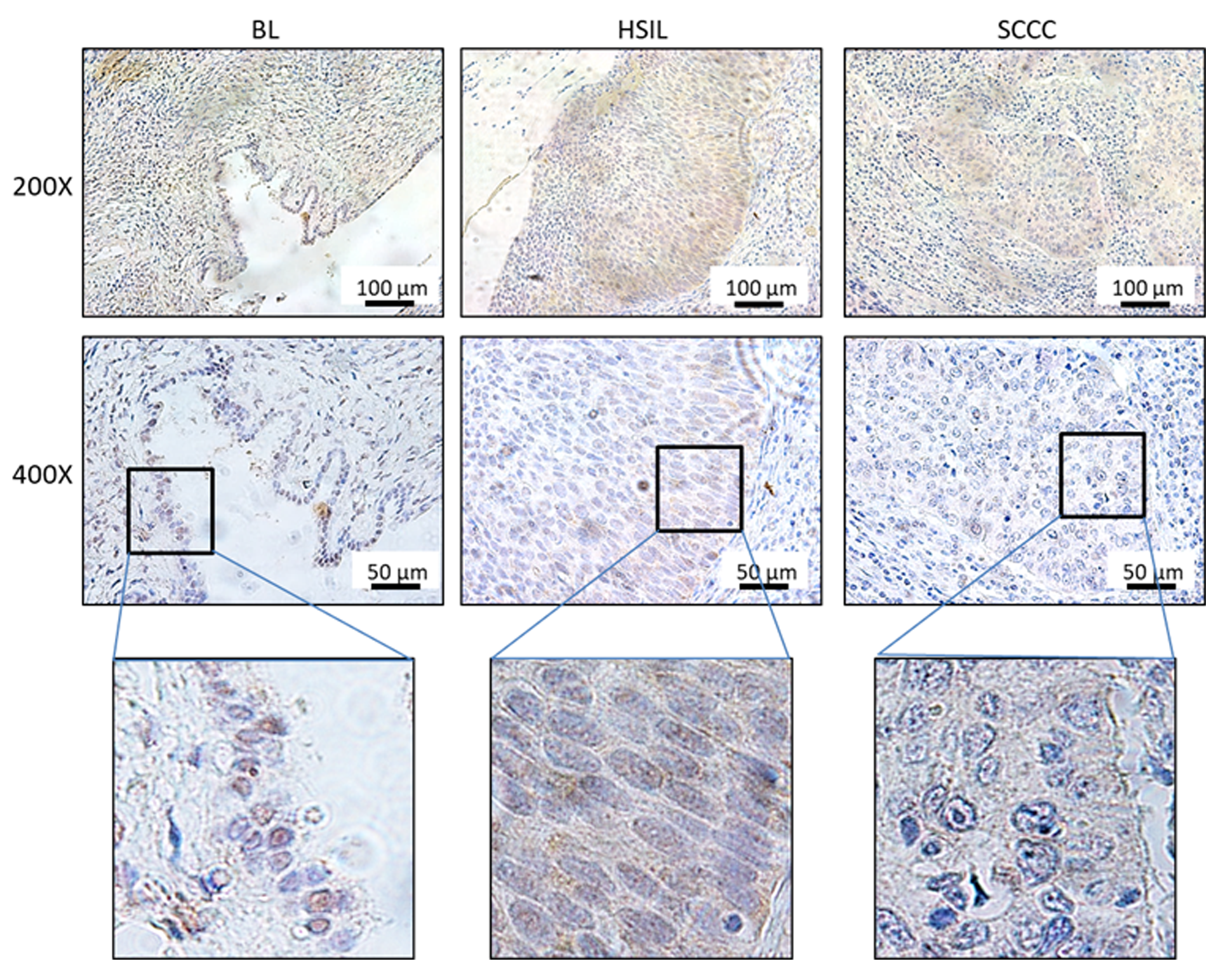

Supplement: Supplementary file 1 [file cancers-13-01739-s001.zip › Supplementary Figure S3.tif]

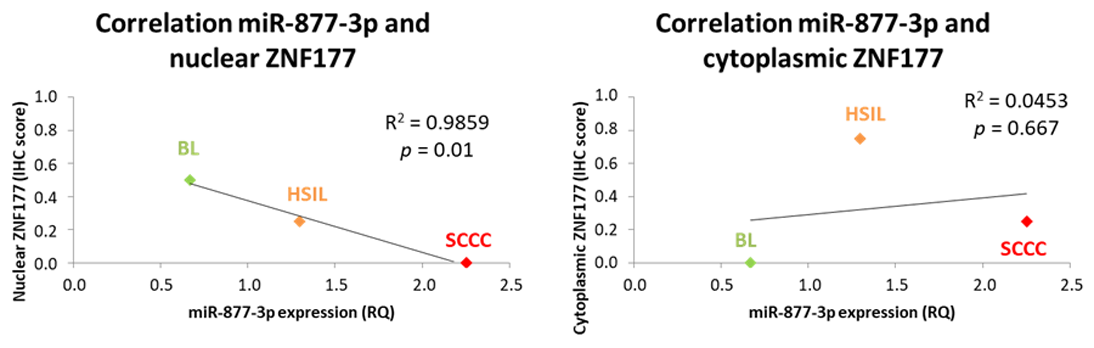

Supplement: Supplementary file 1 [file cancers-13-01739-s001.zip › Supplementary Figure S4.tif]

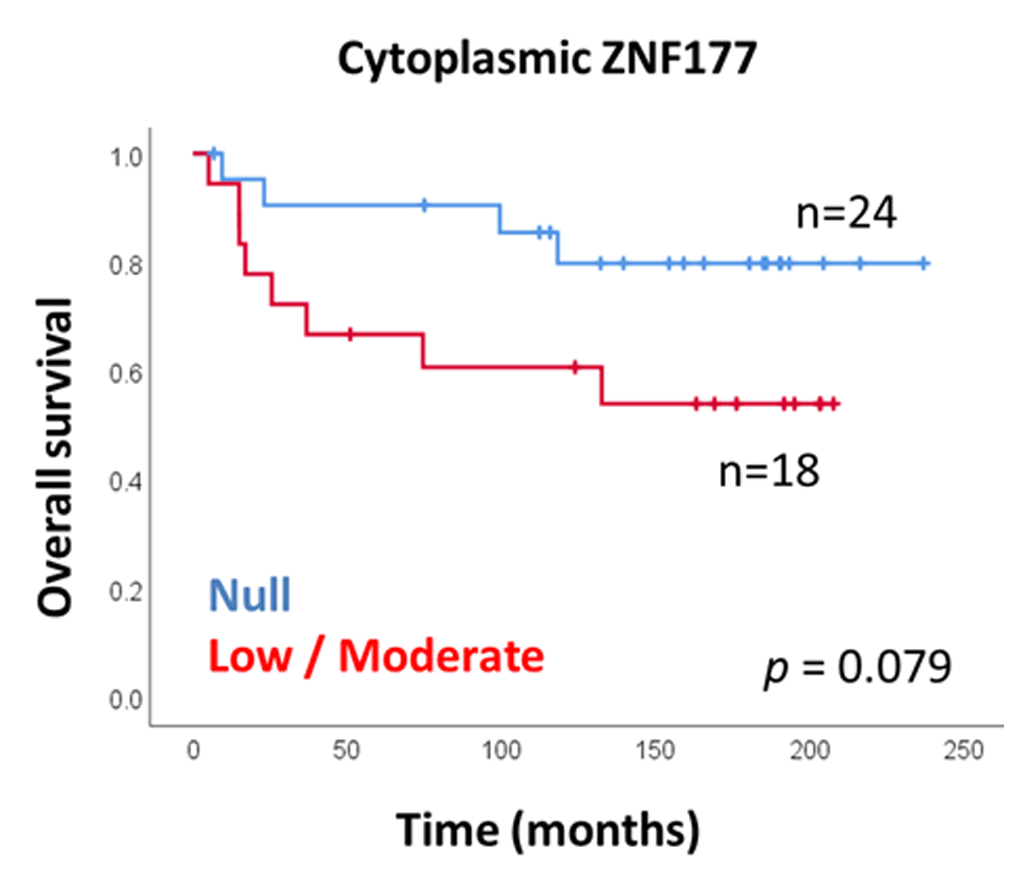

Supplement: Supplementary file 1 [file cancers-13-01739-s001.zip › Supplementary Figure S5.tif]

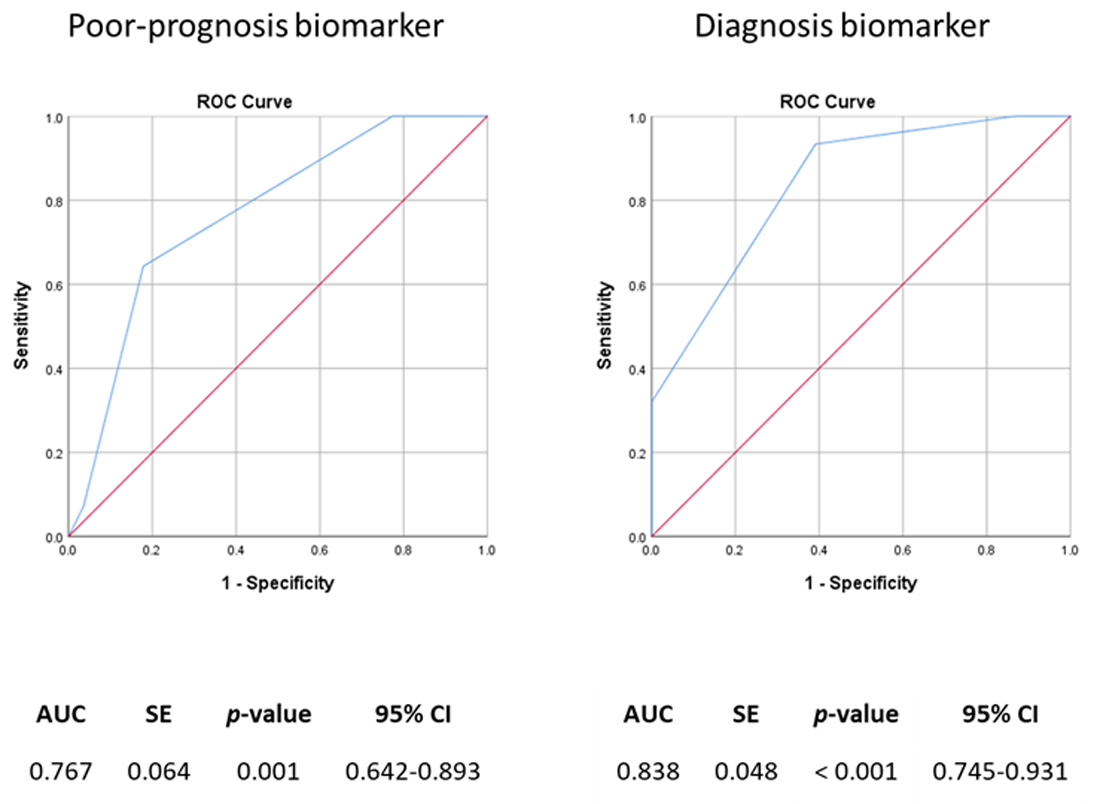

Supplement: Supplementary file 1 [file cancers-13-01739-s001.zip › Supplementary Figure S6.tif]

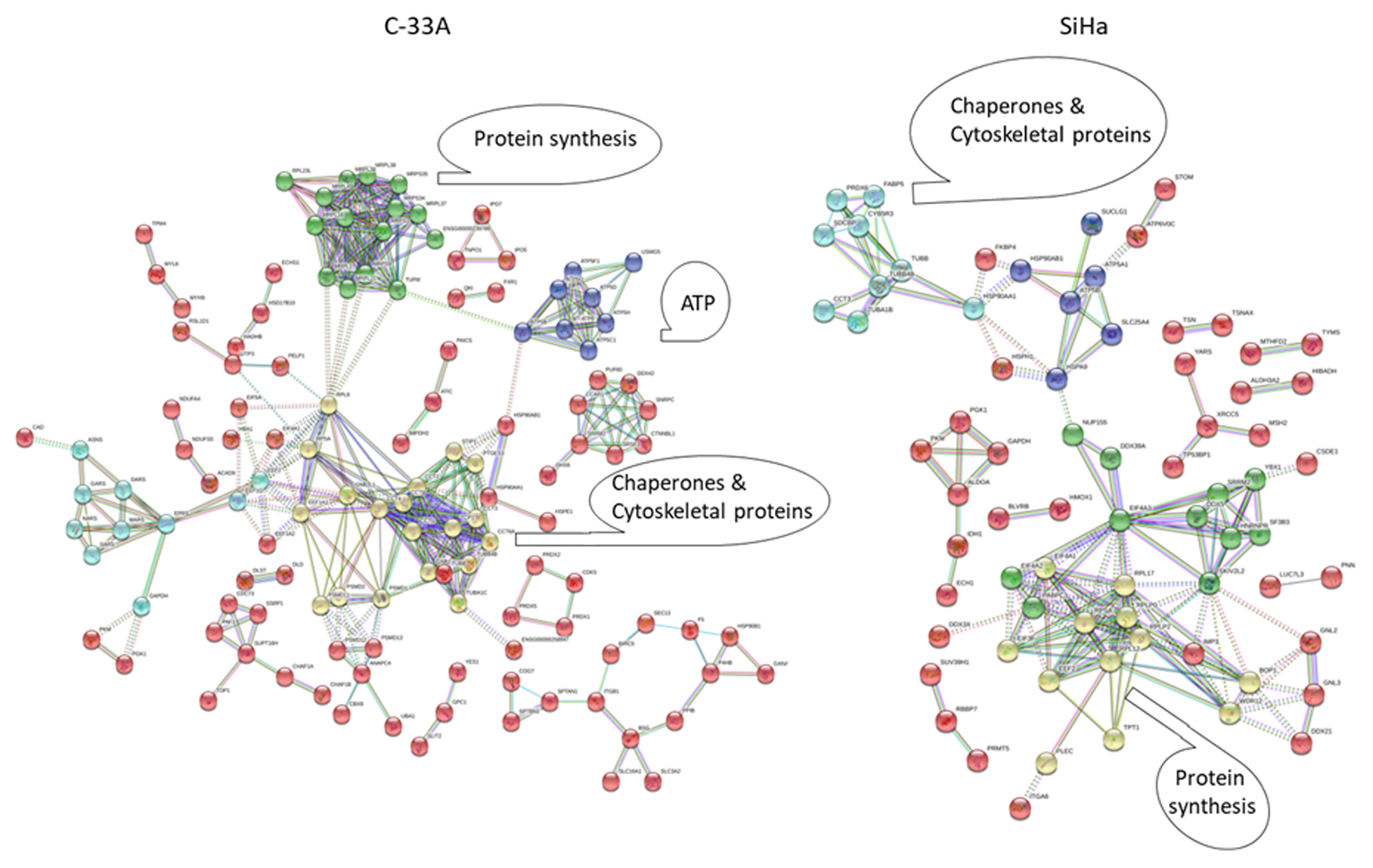

Supplement: Supplementary file 1 [file cancers-13-01739-s001.zip › Supplementary Figure S7.tif]

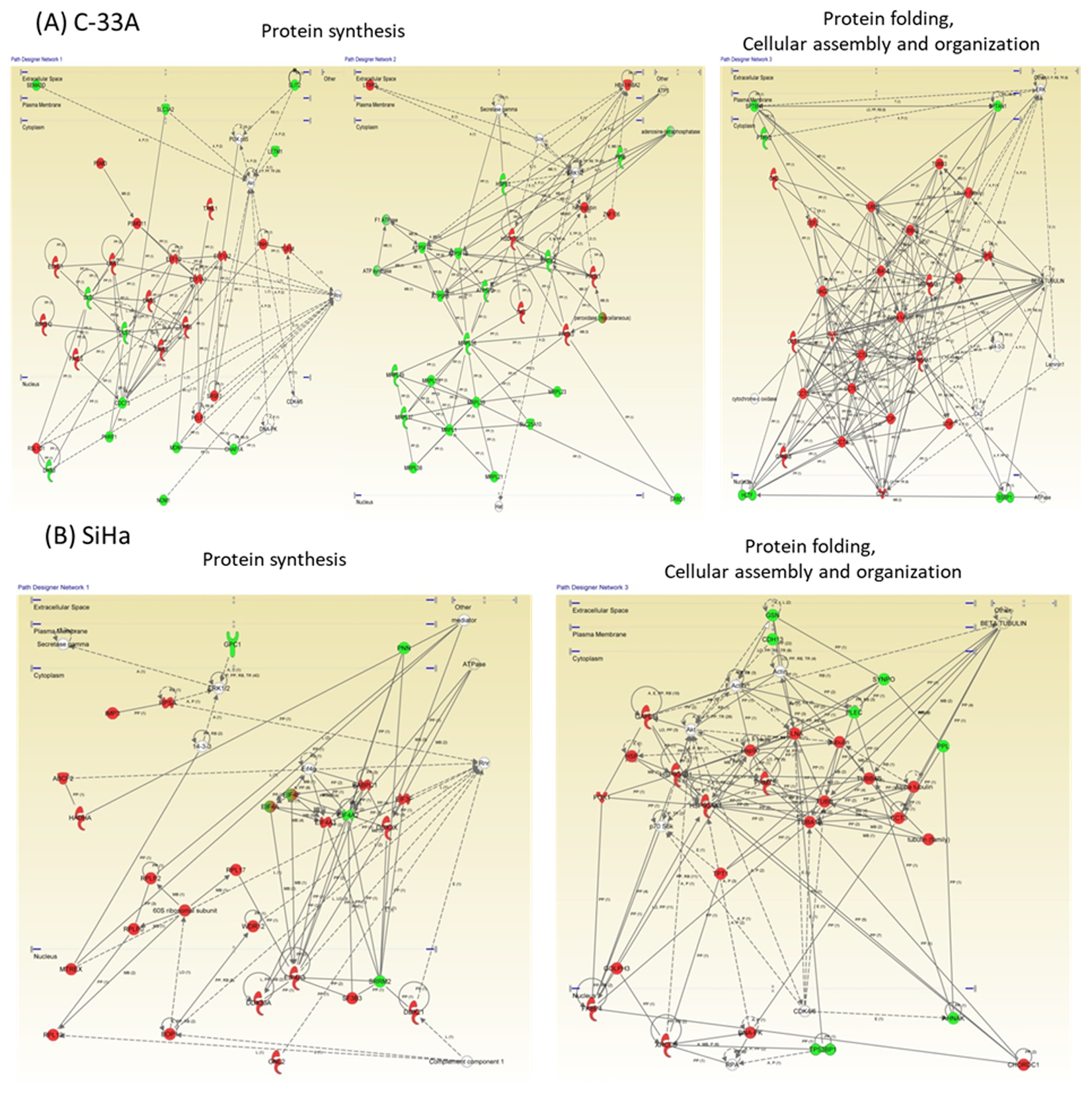

Supplement: Supplementary file 1 [file cancers-13-01739-s001.zip › Supplementary Figure S8.tif]

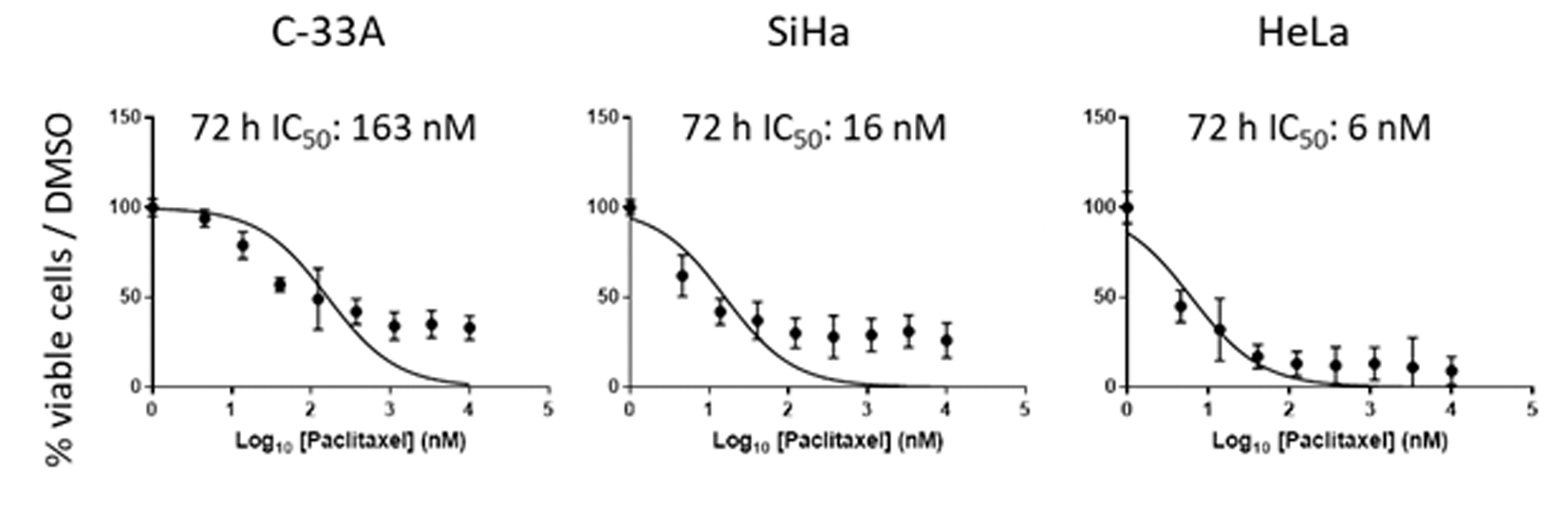

Supplement: Supplementary file 1 [file cancers-13-01739-s001.zip › Supplementary Figure S9.tif]

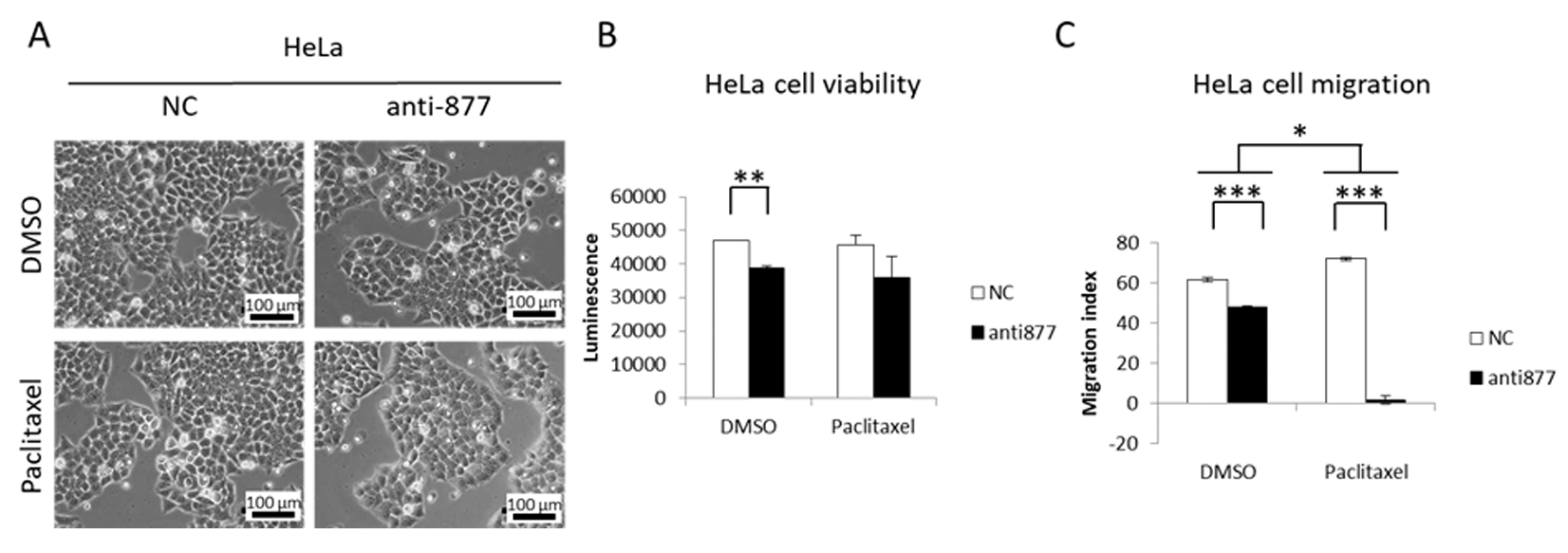

Supplement: Supplementary file 1 [file cancers-13-01739-s001.zip › Supplementary Figure S10.tif]

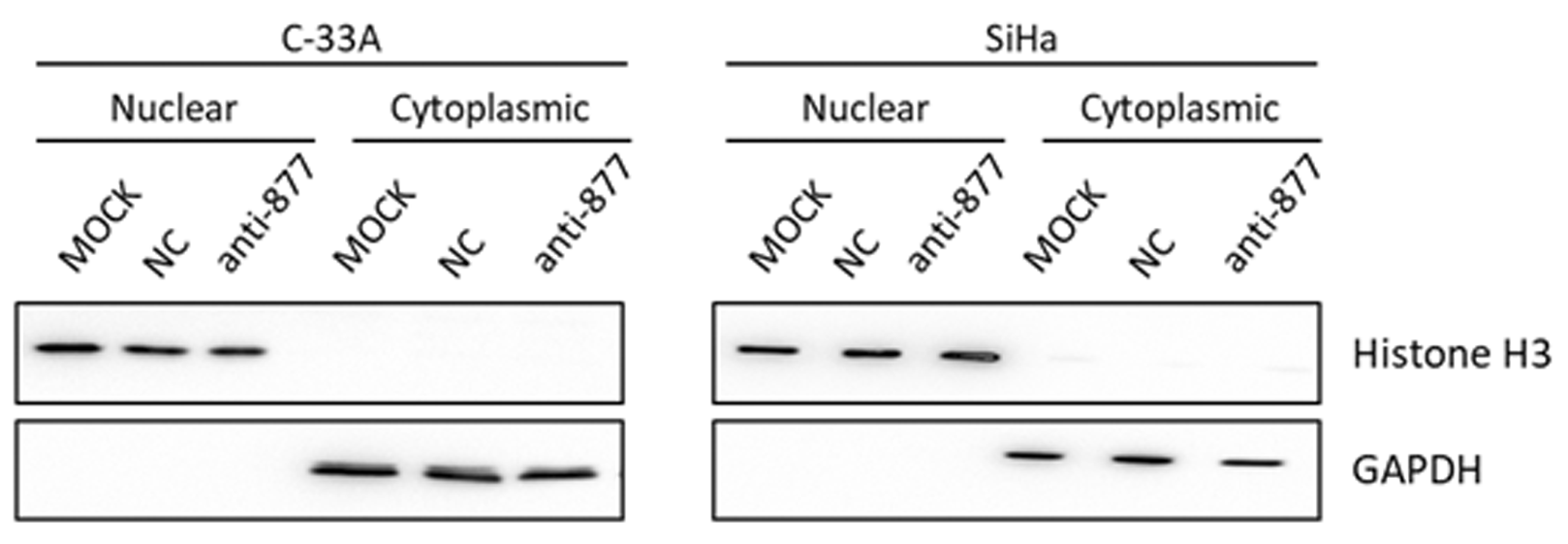

Supplement: Supplementary file 1 [file cancers-13-01739-s001.zip › Supplementary Figure S11.tif]

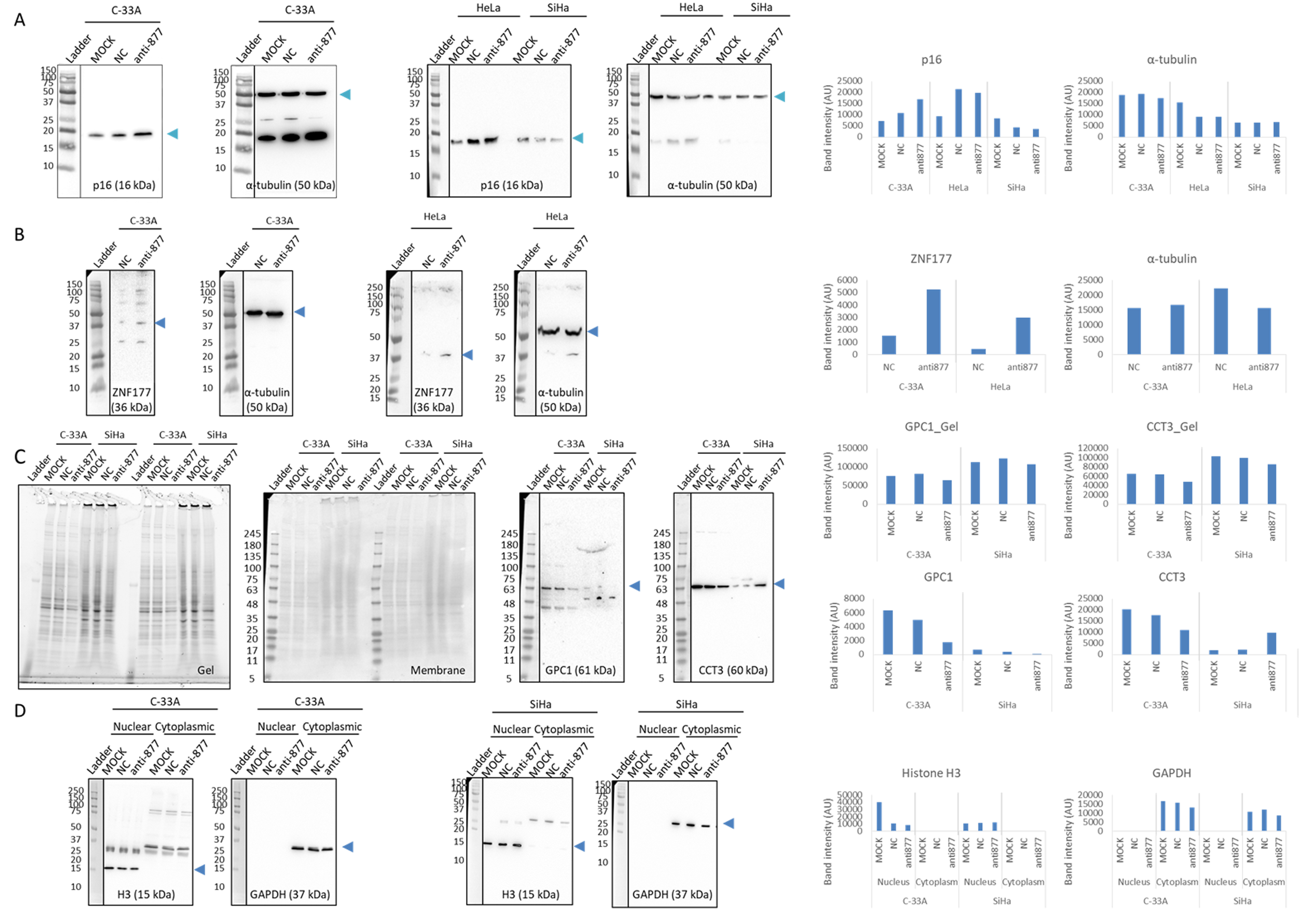

Supplement: Supplementary file 1 [file cancers-13-01739-s001.zip › Supplementary Figure S12.tif]

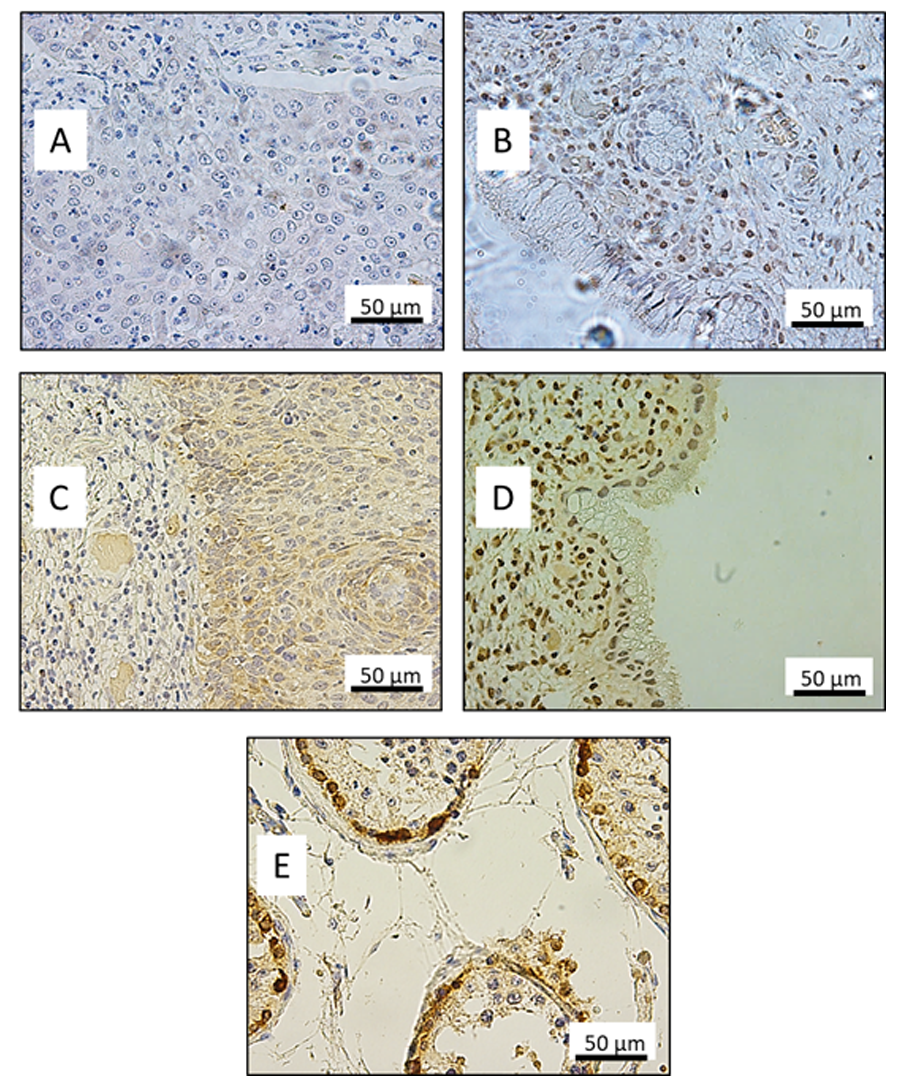

Supplement: Supplementary file 1 [file cancers-13-01739-s001.zip › Supplementary Figure S13.tif]
